# Brain stars take the lead during critical periods of early postnatal brain development: relevance of astrocytes in health and mental disorders

**DOI:** 10.1038/s41380-024-02534-4

**Published:** 2024-03-29

**Authors:** Eugenia Vivi, Barbara Di Benedetto

**Affiliations:** 1 Laboratory of Neuro-Glia Pharmacology, Department of Psychiatry and Psychotherapy, University of Regensburg, 93053 Regensburg, Germany; 2 Regensburg Center of Neuroscience, University of Regensburg, Regensburg, Germany

**Keywords:** Neuroscience, Molecular biology

## Abstract

In the brain, astrocytes regulate shape and functions of the synaptic and vascular compartments through a variety of released factors and membrane-bound proteins. An imbalanced astrocyte activity can therefore have drastic negative impacts on brain development, leading to the onset of severe pathologies. Clinical and pre-clinical studies show alterations in astrocyte cell number, morphology, molecular makeup and astrocyte-dependent processes in different affected brain regions in neurodevelopmental (ND) and neuropsychiatric (NP) disorders. Astrocytes proliferate, differentiate and mature during the critical period of early postnatal brain development, a time window of elevated glia-dependent regulation of a proper balance between synapse formation/elimination, which is pivotal in refining synaptic connectivity. Therefore, any intrinsic and/or extrinsic factors altering these processes during the critical period may result in an aberrant synaptic remodeling and onset of mental disorders. The peculiar bridging position of astrocytes between synaptic and vascular compartments further allows them to “compute” the brain state and consequently secrete factors in the bloodstream, which may serve as diagnostic biomarkers of distinct healthy or disease conditions. Here, we collect recent advancements regarding astrogenesis and astrocyte-mediated regulation of neuronal network remodeling during early postnatal critical periods of brain development, focusing on synapse elimination. We then propose alternative hypotheses for an involvement of aberrancies in these processes in the onset of ND and NP disorders. In light of the well-known differential prevalence of certain brain disorders between males and females, we also discuss putative sex-dependent influences on these neurodevelopmental events. From a translational perspective, understanding age- and sex-dependent astrocyte-specific molecular and functional changes may help to identify biomarkers of distinct cellular (dys)functions in health and disease, favouring the development of diagnostic tools or the selection of tailored treatment options for male/female patients.

## Introduction

Astrocytes are the most abundant subtype of glial cells populating the brain and spinal cord [1]. Microscopically, they show a typical star-shaped morphology with few major processes extending from the soma, which further ramify into numerous fine branches and leaflets at more distal locations [2, 3]. In the past years, several studies and excellent reviews have offered historical overviews about the discovery of astrocytes and covered their numerous physiological roles in the mammalian brain, ranging from their support of the formation/function of neuronal synapses to the development of a properly operating blood-brain barrier (BBB) [4–18].

Here, we collect recent findings on astrocyte-dependent contributions to brain development during early postnatal critical periods, focusing on synapse elimination (phagocytosis) and its putative link to the onset of neurodevelopmental (ND) and neuropsychiatric (NP) disorders. Finally, considering the asymmetry in the prevalence of mental disorders between males and females, we discuss the impact of sex differences on astrocyte properties during critical periods and propose their hypothetical causal relation to sex-skewed brain pathologies.

**Fig. 1:**
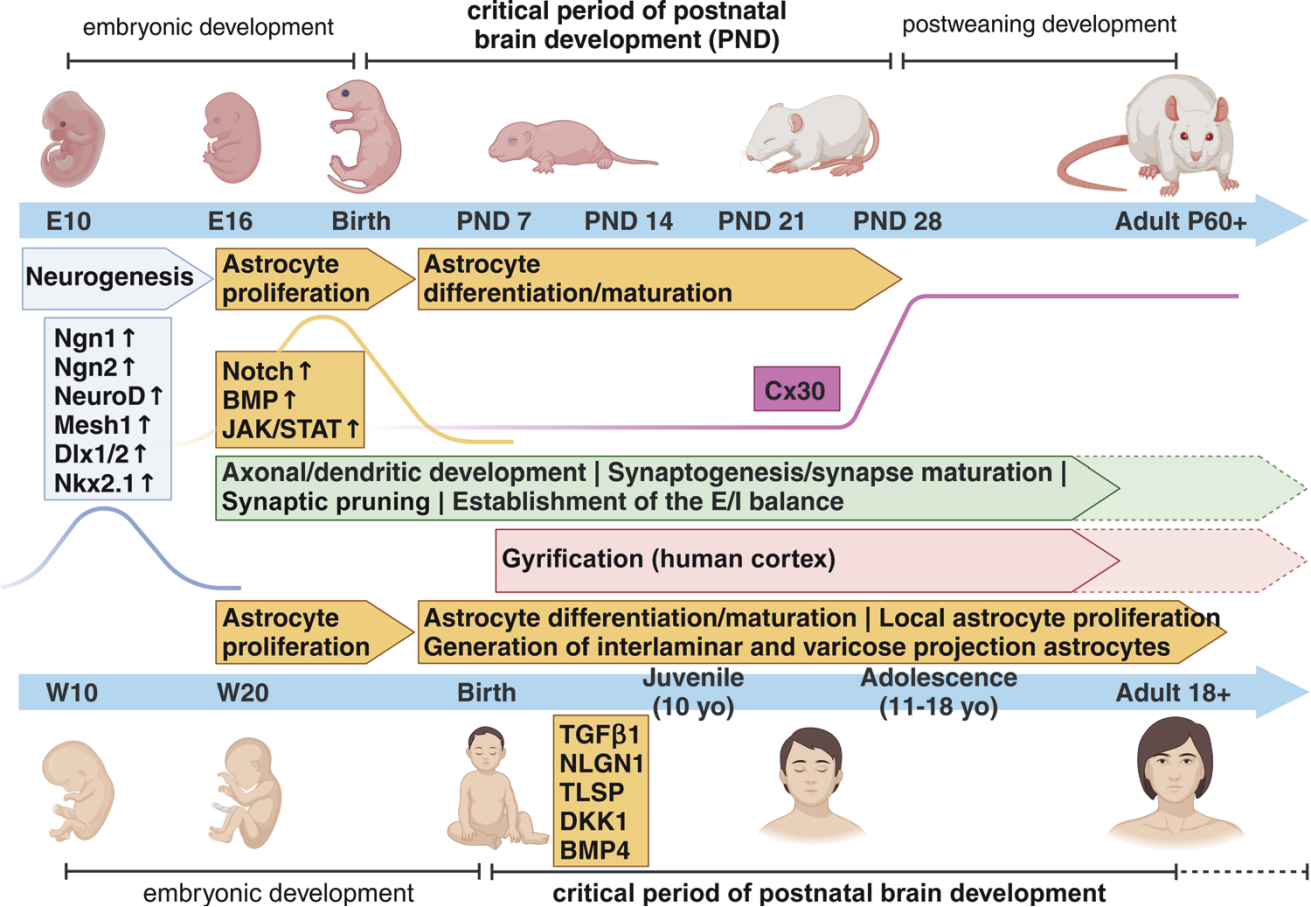
The critical period for astrogenesis and synaptic circuit formation during postnatal brain development in rodents and humans. The timelimes show the major processes occurring from pre- and postnatal early stages through adulthood in rodents and humans to develop properly functional astrocyic and neuronal synaptic networks. A few examples of intracellular signaling molecules which determine the switch between neurogenic to gliogenic cell fates are depicted, as well as selected proteins relevant for astrocyte-specific differentiation/maturation processes (e.g. Cx30 and TGFβ/NLGN1/TLSP/DKK1/BMP4 ligands). We refer the reader to the text for details regarding experimental evidences supporting our current knowledge about each event displayed in the Figure. Cx30, connexin 30. Figure created with BioRender.com.

During embryogenesis, in both human and rodent brains, astrocytes are generated from radial glia (RG), which self-renew and differentiate into neurons and macroglia cells, e.g. astrocytes, oligodendrocytes and Schwann cells [19–25]. Cell divisions of RGs are predominantly neurogenic at early/mid-gestation and turn into gliogenic at late-gestation/early postnatal developmental stages [23, 26, 27] (Fig. 1). The initial steps of gliogenesis produce astrocyte precursors, which then locally proliferate in the different brain areas to increase their numbers and give rise to mature astrocytes, the majority of which consist of protoplasmic and fibrous astrocytes in the gray matter and white matter, respectively [17, 20, 23, 28]. Several studies highlight the heterogeneity of astrocyte morphologies and functions in various regions of the central nervous system (CNS). The most specialized subtypes display very distinctive structural and functional properties such as e.g. the Bergmann glia and velate astrocytes in the cerebellum, the Müller glia cells in the retina or the pituicytes in the neurohypophysis [17, 29–32]. In addition to these general features common to all mammalian brains, hominid primates exhibit evolutionary unique subtypes of astrocytes, i.e. the interlaminar and varicose projection astrocytes [15, 18] (Fig. 1). Interlaminar astrocytes were first observed in Golgi stained samples of the human cortex by Martinotti, Andriezen and Retzius already by the end of the 19^th^ century and described as cells with small somata residing in the upper cortical layer and long processes extending through deeper layers [33–35]. Only later, Colombo and colleagues used glial fibrillary acidic protein (GFAP) to examine these long interlaminar astrocyte processes and proposed that they were primate-specific and putatively involved in favouring a radially acting, long distance intercellular communication across cortical layers [36–39]. A more recent study has, however, challenged the idea of interlaminar astrocytes being primate-specific, and showed their presence in rodent brains, although they appear morphologically more rudimentary than their primate counterpart [40]. Varicose projection astrocytes have been first identified by Oberheim and colleagues in 2009, after examining GFAP-labeled human cortical brain slices [15]. Located in layers 5 and 6, they morphologically resemble protoplasmic astrocytes, but possess less branched short processes and few [1–5] long projecting ones bearing varicosities and extending within single cortical layers, suggesting a tangential range of action [15, 41, 42]. The peculiar enrichment of interlaminar and varicose projection astrocytes in human and higher-order primates, when compared to other lower-order primates, suggests their relevance for evolutionary more refined CNS functions, but this has not been definitively proven yet. However, engraftment experiments of human glial progenitors into mouse forebrains at postnatal day 1 have shown increased synaptic plasticity and learning capabilities of the chimeric adult mice, further strengthening the hypothesis of enhanced supportive properties of human astrocytes for specialized CNS abilities [43]. In addition to these morphological differences, human astrocytes show a remarkable faster propagation of calcium waves when compared to rodents’ astrocytes, in contrast to more evolutionary conserved characteristics, such as an elevated intracellular calcium level in response to ATP and glutamate [15, 43]. For more detailed comparisons between human and rodent astrocytes, we recommend the excellent reviews from Vasile and colleagues [14] and Verkhraktsy and Nedergaard [17].

Beside these differences in subtypes of astrocytes, in various species from worms and insects to rodents and humans, the number of glia cells and the ratio glia-to-neurons are additional factors worth of consideration, because they both increase with the complexity of the CNS [44, 45], suggesting a tight link between the growing number of glial cells with increasingly advanced CNS functions. For instance, the development and evolution of mammalian higher cognitive abilities correlates with the process of gyrification in the cerebral cortex during early postnatal developmental stages [46, 47]. This was shown to be associated with a fibroblast growth factor (FGF)-dependent localized expansion of astrocyte numbers in the ferret cortex, with FGF1 and FGF2 being the predominant ligands involved [48, 49]. Using a targeted genetic manipulation, Shinmyo and colleagues demonstrated that inhibition of astrocyte proliferation in the ferret cortex prevented the formation of gyri, whereas an FGF-dependent overproduction of astrocytes in the lissencephalic mouse cortex was sufficient to induce gyrus-like protrusions [48].

In general, astrocytes show a great range of heterogeneity in the acquisition of various competencies selectively matching the function(s) of neighboring cells [2, 50–52]. To gain such a high degree of intra- and inter-regional diversity, astrocytes must undergo positionally and temporally regulated developmental programmes, strictly modulated by interactions between intrinsic (cell-autonomous) and extrinsic (non cell-autonomous) factors, to ultimately guarantee their specification most aligned to the requirements of the local environment (reviewed in [26]). Among intrinsic programmes, three pathways emerged as crucial players for astrogenesis: the Notch, the BMP, and the JAK-STAT signaling pathways [51, 53–55]. These pathways, alone or in combination with exogenously secreted molecules, e.g. leukemia inhibitory factor (LIF), bone morphogenetic protein (BMP), sonic hedgehog (Shh), ciliary neurotrophic factor (CNTF) or cardiotrophin-1 (CT-1), induce chromatin changes, promoting astrocyte generation and differentiation from RGs [20, 54]. For example, the coordinated activity of Notch and JAK/STAT pathways induces an astrocytic fate by demethylating and thereby activating astrocytic genes, such as GFAP or S100β, with the intermediate activation of the transcription factor STAT3 and the DNA methyltransferase 1 (DNMT1) ([20, 56] and reviewed in [54]). On the contrary, the activity of pro-neurogenic factors such as Neurogenin1 (Ngn1), Ngn2, NeuroD, Dlx1/2, Nkx2.1 and Mesh1 inhibits astrogenesis by directly or indirectly blocking the JAK/STAT pathway, favouring the production of excitatory and inhibitory neurons both in vitro and in vivo [23, 54, 57–69]. Pro-neurogenic factors promote a temporally simultaneous generation of excitatory and inhibitory neurons during embryonic development, but exert different positional influences: excitatory neurons are produced in the dorsal pallium by the radial migration of locally born neuronal progenitors into their targeted cortical layers, whereas inhibitory neurons are generated in the ventral brain and tangentially migrate to dorsal locations, thus infiltrating the developing cortical plate and dorsal areas [70]. These differences influence each other’s developmental trajectories and astrocyte differentiation, ultimately affecting neuronal circuit formation. Imbalances in this finely tuned ratio may contribute to the cognitive dysfunction and neurological abnormalities of mental disorders, such as autism spectrum disorders (ASD), Alzheimer´s disease and schizophrenia (SCZ) [71–73].

Beyond intrinsic factors, evidence that also extrinsic signals guide astrogenesis initially came from co-culture experiments of mouse embryonic RG with cortical slices, which produced neurons when co-cultured on embryonic slices but shifted to a glial fate when co-cultured on postnatal slices [27]. Interestingly, the postnatal release of cytokines such as the gliogenic CT-1 from young neurons promote astrocyte differentiation and their depletion can severely impair astrogenesis [26, 27, 74]. Similarly, in the cerebellum, neuronal Shh diversifies molecular and functional features of Bergmann glia and velate astrocytes [75]. More generally, a coordinated expression of cell-type-specific ligands and receptors is required to favor selected cell-cell interactions and support the appropriate activation of intracellular pathways to drive an astrocyte heterogeneous specification. Very recently, Voss and colleagues demonstrated that the synergistic and combinatorial activity of five ligand–receptor pairs, driven by the ligands TGFβ2, NLGN1, TSLP, DKK1 and BMP4, guides astrogenesis in human cortical organoids and primary fetal tissue. In this work, they additionally identified a time frame of effective responsivity to gliogenic signals, which corresponds to the initial postnatal developmental stages [76] (Fig. 1).

In summary, a tight regulation and coordination of all intracellular and extracellular stimuli along with cell-cell interactions is implicated in controlling astrocyte proliferation and maturation during perinatal and early postnatal developmental periods. This goes hand in hand with neuronal development and is critical for establishing the appropriate subtypes and numbers of astrocytes in any distinct brain region. The balanced relative number of all different cell types, their appropriate functional diversification and the correct formation of elaborated neuronal networks guide the specification of high-order morphological modifications of cortical areas, e.g. gyrification of the cerebral cortex [48, 53, 77]. These are in turn critical for the proper acquisition of increasingly more complex CNS functions, such as human mental and cognitive abilities [1, 44].

Any disruption in these delicate processes might affect the formation and functions of astrocytic and neuronal networks, leading to the onset of severe brain disorders.

## Astrocytes and the critical period of early postnatal brain development in health and mental disorders

All phases of astrogenesis (generation/proliferation, differentiation and maturation) take place with distinct time courses in various areas of the CNS during the so-called “critical periods” of postnatal brain development. These periods may vary both in length and cell types involved in different species, depending on the functions that must be refined [78–81]. Critical periods are also sometimes referred to as “sensitive” periods, although it is still debated whether critical and sensitive periods temporally overlap or should be distinguished [81]. These are time frames when brain plasticity and differentiation/maturation processes are strongly dependent on experience and environmental cues to customize neural circuits and connectivity to the needs of each individual. Therefore, any interaction between intrinsic molecular/biochemical programmes with external factors becomes crucial during critical periods in shaping neuronal circuits to respond with the most adapted behaviors in juvenile, adolescent and adult life [82, 83]. As described by Knudsen [81], during these developmental phases structural changes such as axon elaboration and synaptic modifications are instructed by life experiences across brain areas in various species (reviewed in ref. [84]) (Fig. 1).

In general, identifying the beginning and end of critical periods, as well as isolating the main factors shaping their opening and closing, might support investigations of time frame(s) and mechanisms relevant for an optimal acquisition of selected cognitive abilities and general mental competences, ultimately contributing to build an individual’s personality. From a translational perspective, a better knowledge of the neurobiological, genetic and environmental determinants affecting the opening/closing of critical periods and the reactions of specific cell types upon exposure to selected physiological or detrimental triggers might be essential to identify time windows, when therapeutic strategies are more effective in re-directing aberrant brain developmental trajectories by directly acting on affected cellular mechanisms.

In rodents, astrogenesis occurs during late-gestation and proceeds over the first three weeks of postnatal CNS development, concomitantly with synaptogenesis, both being key processes for the generation of fully functional neuronal networks [84] (Fig. 1). Many astrocyte-derived molecules have been identified so far, playing crucial roles for the formation/functions of excitatory/inhibitory synapses during these developmental time frames. Tan and colleagues have recently reviewed the current knowledge about the cellular and molecular mechanisms by which astrocytes instruct synapse formation/functions through various proteins such as e.g glypicans, TNFα, thrombospondins, Hevin/SPARC ([85–93] further reviewed in [5, 6, 13, 94, 95]). Additionally, a growing body of evidence is featuring the central role of astrocytes in selectively eliminating synapses during developmental windows [96].

In humans, it is still debated whether critical periods are closed after the juvenile developmental stage (approximately corresponding to the first decade of life) or they further extend into adolescence and early adulthood [79, 97]. The initial idea was that the closure of the critical period at the end of puberty marks the end for astrogenesis and synaptic spine formation/elimination in the human cortex. However, this hypothesis mostly relies on the first publications of Huttenlocher and colleagues [98, 99], which contained only one brain specimen to support the claim. In contrast, more recent brain scan studies suggest that several dynamic changes in cortical gray matter density and remodeling extend into the third decade of life [79, 97, 100, 101]. Insofar as dendritic growth and branching are mainly limited to early childhood stages, it has been postulated that this supplementary reorganization of neuronal circuitries is centered on synaptic modifications to favor the acquisition of higher brain functions, such as modulation of emotions, cognitive flexibility, socialization skills and others. In this line, investigating how a proper excitatory/inhibitory (E/I) balance instructs the opening/closing of critical periods may support our understanding of changes in plasticity related to synaptic modifications. Intriguingly, however, in disease states an E/I imbalance might seem to emerge more as a homeostatic compensatory mechanism to abnormal circuit activity, rather than a primary defective mechanism caused by e.g an inherited genetic background [102]. The E/I balance is in fact also heavily affected by neighboring cells, including astrocytes. For example, the deletion of glutamate transporters GLAST and GLT1 in mice results in pathological repetitive behaviors commonly observed in ASD, obsessive-compulsive disorder, and Tourette´s syndrome [103]. Moreover, one study showed that astrocyte reactivity reduces inhibitory currents as a consequence of diminished glutamine and GABA availability [104], although astrocyte reactivity may not always homogeneously respond to disease conditions [105].

Besides these evidences, other theories to explain late-onset neurological or NP disorders suggest defective pruning mechanisms of the initially supernumerary spine synapses among the causes of disease conditions [106–109]. An over-abundance of unpruned weak synaptic inputs appears to negatively affects the synchronized development of E/I inputs, ultimately disrupting the connectivity of brain circuits in the limbic system [13, 110, 111].

However, due to the limitations of human neuroimaging tools and the ethical issues associated with studies involving children and adolescents, understanding the cellular mechanisms behind these developmental periods and how perceived adverse events may become neurobiologically embedded in brain circuits and result in ND and NP disorders, requires the continuous development of experimental models recapitulating the human conditions.

The use of animal models has been pivotal in revealing essential physiological underpinnings of critical periods and unraveling molecular pathways that respond to the simultaneous application of pharmacological treatments together with behavioral interventions, to ultimately rewire altered neuronal circuits [112–114]. Few seminal studies examined changes in the plasticity of brain circuits in various brain regions, after the application of effectors aimed at reopening critical period-like states. Specifically in two of them, the chronic administration of the antidepressant fluoxetine showed remarkable effects on the reopening of critical period-like neuronal responses, further associated with phenotypical alterations. Interestingly, in a first study, fluoxetine reinstated an ocular dominance plasticity in adult mice, promoting the recovery of visual capabilities in amblyopic animals, accompanied by a reduction of intracortical inhibition [113]. A later work showed how fluoxetine treatment combined with behavioral extinction training induced the erasure of amygdala-dependent fear memories, after reactivating a juvenile-like circuit plasticity associated with a disruption of extracellular matrix structures called perineuronal nets (PNNs) around parvalbumin-positive interneurons [112].

Although both studies evidenced a “rejuvenalization” of brain circuits with changes in the inhibitory system concurrent to the reopening of a critical period-like plasticity, neither of them proposed a cellular or molecular mechanisms possibly involved in the observed disruption of PNNs. Only very recently, the study of Ribot, Breton and colleagues has highlighted the role of astrocyte to close the critical period for visual plasticity in the mouse, favouring a remodeling of the extracellular matrix and associated maturation of the inhibitory system in the visual cortex, although the excitatory system was also, even if minorly, modulated [114]. Based on the knowledge that in rodents astrogenesis occurs during the early postnatal developmental stages and that it is for the most part completed by the end of the critical period around postnatal day (P) 28, the authors postulated that the maturation of astrocytes and its molecular regulators might be relevant for the closure of this period. In line with this, a previous study showed that transplanting immature astrocytes in the visual cortex of adult cats reactivated a period of high brain flexibility, with associated re-induction of ocular-dominance (OD) plasticity in adult animals, similar to what observed upon fluoxetine treatment [115]. In their study, Ribot, Breton and colleagues demonstrated that a molecular switch from proliferative to mature astrocytes corresponded to the closure of the critical period and the astrocyte-specific gap-junction channel subunit connexin 30 (Cx30), an important modulator of hippocampal astrocyte maturation [116], was a pivotal player in these events (Fig. 1). Its lower expression in immature astrocytes and higher expression in mature astrocytes correlated with different stages of neuronal network maturation and plasticity in the visual cortex. Furthermore, the astrocyte-specific knockdown of Cx30 favored a hightened ocular dominance plasticity until P50, confirming the astrocytic Cx30 as a factor necessary to mark the closing of the critical period [114]. It would be intriguing to investigate whether and how immature astrocytes might characterize ND or NP disorders and potentially identify underlying molecular determinants, which might trigger the onset of astrocyte-dependent brain disorders. In humans, early-life adversity (ELA) has been associated with the appearance of severe neurological and mental symptoms later in life, as well as with aberrant astrocyte functions ([82, 117] and reviewed in ref. [84]). Besides ELA, other chronic stress paradigms are accompanied with reductions in both the number of GFAP-positive astrocytes and the length of GFAP-labeled processes in the prefrontal cortex (PFC), hippocampus or other brain regions, besides changes in the expression levels of various astrocyte-specific molecular markers, suggesting a negative impact of stress on proliferation/maturation processes of astrocytes [118–124]. For example, three weeks of mild chronic unpredictable stress reduce the number of Sox9/S100ß double positive cells in different subfields of the hippocampus [125]. Moreover, chronic social defeat stress (CSDS) hampers the expression of glucocorticoid receptors (GR) asymmetrically in astrocytes and neurons of the medial PFC. The reduced GR content in astrocytes sequentially affects Ca^2+^ oscillations and the release of adenosine triphosphate (ATP), which in turn modulates synaptic communication [126]. Additionally CSDS decreases the expression of Cx30 and Cx43, correlating with an impaired frequency of spontaneous EPSCs in slices from the mPFC and hippocampus and suggesting a reversal of the system to a functionally immature phenotype [127]. Furthermore, early social isolation in rodents induces an astrocytic GR-dependent activation of MERTK pathway, ultimately leading to an excessive phagocytosis of excitatory synapses and consequent unbalanced neuronal firing patterns [128]. Strikingly, a selectively lower expression of the astrocyte-specific protein Cx30 characterizes postmortem brain tissues of patients with major depressive disorder (MDD) [129], supporting a link between the expression of cell-type specific markers reminiscent of a critical period-like brain state and CNS pathologies. Furthermore, worth to mention is the association of several mental disorders with a reduced gyrification index (GI) in cortical areas, measured by magnetic resonance imaging (MRI) in patients, also suggestive of hypothetical deficits in astrocyte proliferation affecting cortical formation being behind certain brain diseases ([130–132] and reviewed in refs. [133, 134]).

**Fig. 2:**
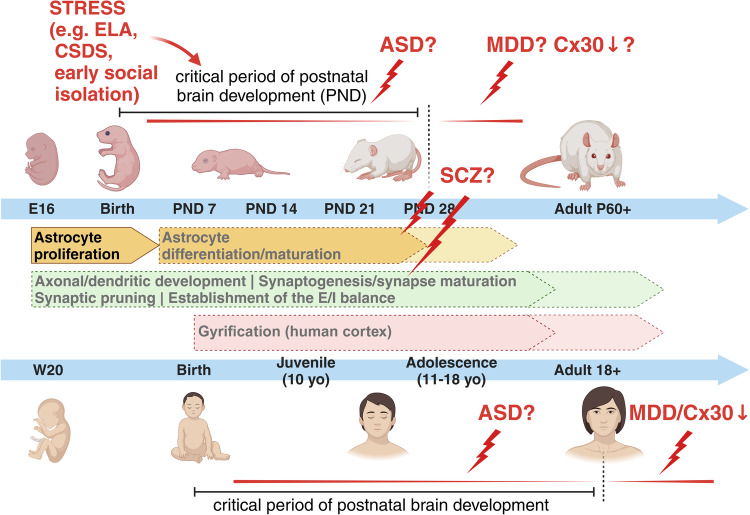
The role of critical period for the onset of neurodevelopmental and neuropsychiatric disorders. Intrinsic molecular programmes and environmental factors may interact with each other during the critical period of brain development to drive the proper formation of neuronal circuits. Any alterations in the sequences of events occurring during these time frames might lead to the onset of neurodevelopmental (ND) disorders such as Autism Spectrum Disorder (ASD) or neuropsychiatric (NP) disorders such as Schizophrenia (SCZ) or Major depressive disorder (MDD). Stressful triggers, such as early-life adversity (ELA), chronic social defeat stress or early social isolation, experienced during the critical period show a higher impact on the development of ND and NP disorders later in life. Molecules like Cx30 may play a role in the closure of the critical period of astrocyte development, which is necessary for the proper formation of brain circuits. Figure created with BioRender.com.

**Fig. 3:**
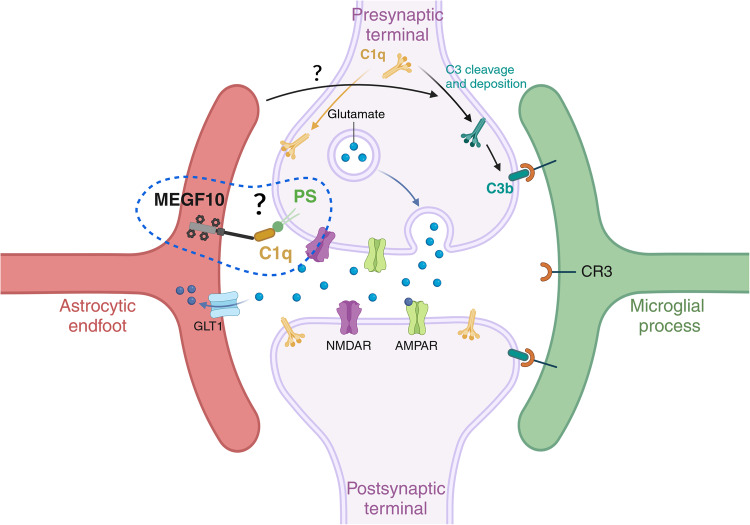
Glia-mediated synaptic pruning. Astrocytes and microglia cells both regulate the refinement of synaptic neuronal networks through the elimination of weak synapses during early postnatal developmental stages. Signaling molecules called “eat me” signals, such as C1q and phosphatidylserine (PS), recruited during these events have been identified, as well as binding partners located on the respective cells, like MEGF10 receptor on astrocytes and the receptors of the complement cascade on microglia cells. It remains unclear whether PS and C1q are directly implicated in the astrocyte-mediated synaptic elimination as well. Figure created with BioRender.com.

Several studies have revealed astrocyte deficiencies in other brain disorders besides MDD, such as SCZ, bipolar disorder (BD), ASD and epilepsy [135–144]. In young MDD patients, postmortem brains present a profound reduction in the number and morphology of GFAP- and S100β-positive astrocytes in the prefrontal and anterior cingulate corteces and in the hippocampus, all regions heavily affected in MDD [135, 138, 145–148]. To validate this, it was also shown that antidepressants target astrocytes and reverse disease symptoms [136, 149–151]. It remains to be clarified, however, which subtype(s) of astrocytes are affected, as far as currently available markers cannot discriminate among interlaminar, varicose projection or protoplasmic astrocytes. Conversely, in SCZ or BD, changes in astrocytes are less consistent, with different studies showing both their increased and decreased numbers in postmortem tissue of SCZ patients [140, 141]. The most reproduced findings show a hypofunction of NMDA receptors (NMDAR) in SCZ. Astrocytes enhance NMDAR activity through the release of gliotransmitters, such as D-serine, which acts as co-agonist of glutamate [152]. The binding of D-serine to synaptic NMDARs additionally modulate the induction of long-term potentiation (LTP), the latter being the cellular correlate of memory formation [153]. Memory deficits are among the most common symptoms of various ND and NP disorders, thereby supporting an involvement of gliotransmission in their etiopathogenesis. In line with this hypothesis, a disrupted NMDAR function may also rely on a imbalanced postsynaptic AMPA receptor composition or missed AMPA receptor maturation due to an altered astrocyte-dependent glypican secretion or chordin-like secretion, respectively [90, 154]. Related to this, the work of Caldwell and colleagues recently showed how an aberrant secretion of astrocyte-derived proteins is associated with severe ND disorders accompanied by an altered neuronal development [155]. Indeed, recent clinical work has proposed D-serine as a promising therapeutic option for the treatment of ND disorders, most notably SCZ [156, 157]. Moreover, it has further been shown that mutations in Disrupted-In-Schizophrenia 1 (Disc1) reduce the stability of the D‐serine synthesizing enzyme serine racemase, resulting in SCZ‐like behavior [158]. Thus, in SCZ, not the number but rather the functionality of astrocyte seems to be mostly affected and influence disease onset and/or progression, principally driven by genetic factors. This might explain the inconsistent results from postmortem brain tissues, which did not show reproducible deficiencies in astrocyte counts [140, 141]. Despite SCZ having been considered a disorder with a high genetic component, in reality the concordance rate among identical twins is only about 50% [159], with a substantial proportion of SCZ being of idiopathic origin. More interestingly, cortisol levels, an index of stress, measured in adolescents at-risk for SCZ were significantly elevated in subjects who transitioned to psychosis later in life, further supporting the negative impact of early life environmental factors such as stress for worsening NP disorders when encountered during critical periods of postnatal development [160, 161]. Although a defective neurogenesis during the critical period has been long described in SCZ and associated with its etiopathogenesis [162], recent work has elucidated the putative role of astrocytes and their aberrant maturation paths impacting synapse formation during critical periods as a possible cause of disrupted neuronal circuit formation and disease onset [142, 163]. In ASD, astrocyte involvement in its etiology has been recently investigated using patient-derived iPSCs [144, 164]. Even in ASD, the essential role of the critical period has been proposed, with a shortened time frame of neuroplasticity indicated as a common cause of disease among various patients, despite their highly divergent genetic backgrounds. In particular, the language and social skill deficits observed in ASD have been led back to a premature closure of the critical period for their development, which should typically extend into adolescence. The hypothesis is that a shorter critical period may force the brain to rely on underdeveloped neuronal networks for learning language and social skills, among other mental abilities, leading to the typical symptoms observed in ASD patients [165]. However, as of today, no work has thoroughly investigated the possibility that an earlier closure of critical periods, marked by the premature differentiation of astrocytes, might be among the neurobiological underpinnings in the etiopathogenesis of ASD. In the same line, no work has ever considered a failure in the closure of this period among possible causes for other mental disorders such as depression (Figs. 2 and 3).

## Astrocytes and the synaptic compartments: the relevance of an astrocyte-mediated phagocytosis for neuronal circuit refinement in health and disease

In the CNS, the peculiar position of astrocytes between synaptic and vascular compartments supports the formation of a functional unit (neurovascular, NVU) “sensing” the brain state and secreting factors in the bloodstream as a reflection of this state. In pathological conditions, such factors may serve as biomarkers of cellular (dys)functions and help to improve diagnostic/treatment options specifically adapted to the needs of individual patients. Several excellent reviews have already described secreted astrocyte-derived molecules relevant for synapse and BBB properties in health and disease [5, 6, 10, 12, 13, 166–169]. We also validated and characterized astrocyte and BBB deficits in an animal model of MDD [168, 170] and identified the astrocyte-derived factor GDF15 as an effector of fluoxetine to simultaneously restore the disrupted astrocyte processes and loosened tight junctions between endothelial cells [171]. Ideally, identifying cell-type specific molecular underpinnings of disease may support the discovery of equally specific diagnostic biomarkers. However, ND and NP disorders often share overlapping symptoms and underlying neurobiological alterations, making it difficult to isolate disease-specific biomarkers for personalized medicine. Therefore, only a combination of efforts and methodological approaches may offer valid guidance to reach this ambitious goal. Among less explored functions of astrocytes, we focus here on astrocyte-mediated synaptic phagocytosis during critical periods of brain development and its putative involvement in NP and ND disorders.

Astrocytes derive from the embryonic ectodermal sheet and therefore share some developmental, genetic and functional similarities with other ectodermally-derived cells, such as neurons and oligodendrocytes, but less with other brain cells of mesodermal origin, such as microglia cells [172]. However, they might adopt analogous functions to microglia cells to cooperatively converge their efforts in the regulation of neuronal networks formation [96, 173].

Together with microglia, astrocytes contribute to the formation/function of excitatory and inhibitory synapses and to the elimination of weak synapses (pruning) through released factors or membrane-bound molecules [51, 169, 173, 174].

Early studies in the fly *Drosophila melanogaster* opened the field of glia-mediated phagocytosis, showing how this process is highly conserved across species, even when species-specific cellular and molecular mechanisms diverged along evolution to adapt to the growing complexity of selected functions [44]. In *Drosophila*, it was shown that, during its larvae-to-pupa metamorphosis, a high degree of tissue digestion occurs to reshape its body structures and nervous system, allowing the subsequent extension of adult, mature, neuronal projections [175]. The sequence of events leading to such drastic changes follows a developmentally regulated pattern, initiated by complex interactions between intrinsic and extrinsic factors, such as the neuronal hormone ecdysone. Specifically, the gene products of Draper and CED-6 were first identified to mediate the engulfment of pruned axons by glia cells during metamorphosis [176]. Accordingly, mutations in these two genes and glia cell-targeted knockdown experiments, suppressed glia-mediated axon pruning during metamorphosis. Genetic studies in *Caenorhabditis elegans* initially isolated the genes encoding for CED-1 and CED-6 as also essential mediators for the clearance of apoptotic cells [177].

In the mouse brain, the seminal work of Stevens [178] and Chung [174] has been key to underscore novel roles for the classical complement cascade and the MEGF10/MERTK proteins, respectively, for the refinement of neuronal synaptic networks during postnatal developmental periods. Initially, microglia were described as key players to refine synapses by engulfing presynaptic inputs during the peak retinogeniculate pruning *via* the microglial CR3/C3 phagocytic signaling pathway and under the regulatory influence of neuronal activity [179]. The involvement of microglia in synaptic pruning was simultaneously uncovered in the hippocampus and juvenile visual cortex, where the synaptic remodeling occurs through the fractalkine/CXC3CR1 signaling pathway [180]. Afterwards, numerous studies and comprehensive reviews have extensively examined the crucial role of microglia cells in the process of synapse elimination and in disease states [181–189]. Later, an additional involvement of the astrocytic MEGF10 and MERTK was highlighted for the synaptic remodeling during postnatal brain development [174] and the maintenance of hippocampal homeostasis in the adult brain [190]. Microglia and astrocytes collaborate through a synergistic and precisely orchestrated spatiotemporal coordination, ensuring efficient homeostatic phagocytosis of apoptotic cells and synaptic pruning, while respecting each other´s territorial boundaries and competences: microglia exhibit an engulfing preference for large cell bodies, whereas astrocytes favor small dendritic apoptotic bodies [191]. Moreover, Lee and colleagues demonstrated how astrocytes and microglia constantly engage in phagocytosis of both excitatory and inhibitory synaptic elements within the hippocampal CA1, preferring excitatory rather than inhibitory terminals [190]. Similarly, Dejanovic and colleagues observed that astrocytes preferentially engulf excitatory synapses, while microglia tend to target inhibitory ones, revealing a divergence between astrocytes and microglia during (patho)physiological processes ([181] and reviewed in ref. [169]). A recent study has identified a unique GABA-receptive microglia subtype that selectively remodels inhibitory synapses during mouse postnatal cortical development [182]. Furthermore, the MERTK-mediated microglial phagocytosis is crucial for eliminating inhibitory post-synapses in the juvenile brain [183]. Elucidating the complex interplay between microglia and astrocyte regulatory roles in synapse elimination is the current crucial focus of many research labs. Regarding the molecular mechanisms driving these events, the current view postulates that a balanced distribution of “eat-me signals” and “don’t eat me signals” is key to determine which cells (or parts of them) should be excised/phagocytosed [51]. Among “eat-me signals”, the C1q has been described multiple times as a common tagging system shared by microglia cells and astrocytes to identify their targets. For instance, MEGF10 can mediate its phagocytic functions on apoptotic material via the recognition of C1q bound to phosphatidylserine (PS) exposed on dying cells [109]. This suggests that regulatory mechanisms controlling C1q expression/tagging and/or its binding partners may be responsible for sorting out which cell type between microglia cells and astrocytes should be recruited to execute the job. Because of the commonly shared target(s), it has also been proposed that the selective mechanism determining which cell type acts first may rely on the specific set of receptors localized on their membranes, possibly activated alone or in combination with other molecules “on demand” [96].

From the literature, the current hypothesis is that astrocytes stimulate neurons to produce C1q, which then triggers the downstream activation of the complement cascade and C3b deposition, finally promoting synapse elimination through its microglia-specific binding partner C3 receptor (CR3) [178, 179] (Fig. 3). Another extensively studied “eat-me signal” in the microglia-mediated synaptic elimination is represented by PS exposure on neuronal membranes. The PS presentation on weak synapses promotes microglia-mediated synaptic pruning via the TREM2 receptor and C1q in the developing hippocampus and retinogeniculate system [184]. Beside these positive mediators of microglial engulfment, “don’t eat me signals” also modulate synaptic pruning. These signals counterbalance the effects of “eat me signals”, preventing the engulfment of viable synapses by aberrant microglial removal. Among them, CD47 was first characterized as “don’t eat me signal” because it permitted glioma cells to infiltrate the healthy brain parenchyma, after blocking their macrophage-mediated engulfment [192]. Its alternative function in synaptic pruning was uncovered later, with CD47 expressed on neuronal membranes and interacting with the microglial receptor SIRPα being responsible for suppressing synaptic phagocytosis. Mice lacking CD47 exhibit excess synaptic pruning and decrease synaptic connectivity [193], whereas its increased expression was found in iPSCs derived from autistic patients with enlarged brains accompanied by reduced cellular phagocytosis [194]. However, in contrast to the extensive literature describing the mechanisms of a microglia-mediated synaptic pruning in health and disease [178, 179, 195, 196], those instructing the astrocyte-mediated synaptic elimination are still less known [96, 128, 173, 174, 190] (Fig. 3).

A reduced proliferation of astrocytes and/or disruption of astrocytic activity characteristic of ND and NP disorders may affect the appropriate formation of synaptic circuits in different ways. Their impaired activity in pathological conditions may for example either weaken mature synapses or prevent their maturation, thereby potentially enhancing their susceptibility to an excessive pruning. Alternatively, an aberrant astrocyte activity may negatively influence the synaptic tagging, leading to either an excessive or abrupted elimination of unwanted synapses. These possibilities further sustain the importance of the critical developmental periods, when astrocyte proliferation/maturation and synaptogenesis take place simultaneously, in sculpting brain circuits in health and disease.

## Sex-dependent differences in astrogenesis and in time frames of vulnerability to perturbations and disease onset

Sex differences have long been recognized as a variable affecting many brain disorders in terms of predisposition, rates of incidence, age of onset, symptomatology and outcome, with skewed prevalence towards one sex in different pathologies [111, 119, 197–199]. However, research on sex-dependent disorders and their neurobiological molecular and cellular causes is still scarce, thereby limiting the development of sex-specific diagnoses and treatments.

**Fig. 4:**
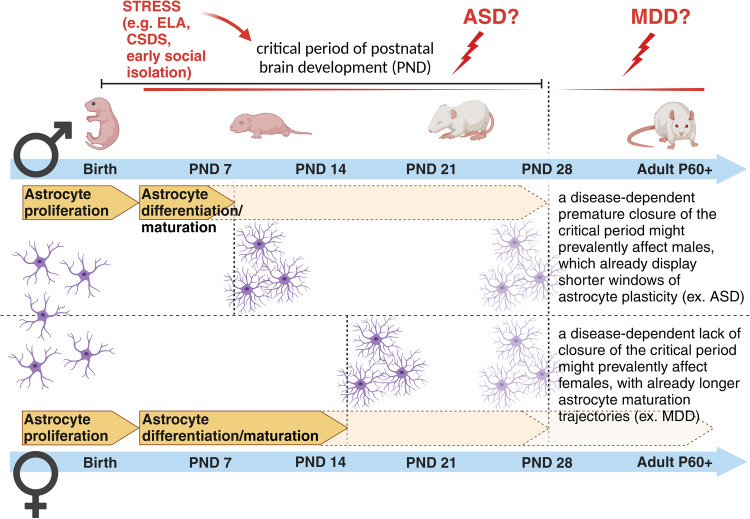
Sex-dependent differences in astrogenesis and vulnerability to disease onset. A sex-dependent predisposition to brain disorders has been long recognized for many brain pathologies. Differential time-shifted maturation trajectories observed in astrocytes and astrocyte-mediated processes between males and females may account for such sex-dependent biased windows of vulnerability to disease onset. Figure created with BioRender.com.

In the past, many human studies have often lacked sufficient sample availability to stratify results based on sex differences for any given disease category. Nowadays, the access to huge genomic, transcriptomic and proteomic datasets help to discriminate the impact of sex variables on various biological parameters [200–203]. However, our understanding of whether and how sex might affect astrogenesis, synaptogenesis and synapse elimination in humans and to which extent this contributes to the onset of brain diseases is still limited [204]. Currently, the overall idea is that mental conditions with early-onset neurodevelopmental origin, such as ASD, attention deficit/hyperactivity disorder and SCZ show a higher male prevalence. On the other hand, disorders with a higher emotional component, such as depression, anxiety disorder, and eating disorders, which usually start during puberty or later in life, show a higher female incidence [205, 206]. This asymmetric development of psychopathologies has been analyzed across studies with the goal of elucidating how and which sex-specific developmental maturation trajectories might influence it. Interestingly, regional differences in volume and tissue density were found between sexes in areas implicated in sex-biased NP conditions, suggesting a few candidate regions susceptible to sex differences in the developing brain [207]. Among those regions, the cortex has been implicated as one of the brain areas with a high sex-dependent diversification in cellular and synaptic densities [99, 111, 119, 208]. Most of these differences have been attributed to remodeling processes occurring at the synaptic level, with an aberrant pruning of weaker synapses being recently regarded as a mechanism relevant for the onset of brain disorders [110, 111]. As previously mentioned, most of these remodeling events take place during the critical periods of brain development, when astrogenesis in parallel with synaptogenesis contribute to the formation and refinement of neuronal circuits [54, 78, 79, 209]. Mechanisms controlling the opening and closure of these developmental time windows might be essential in building healthy neuronal networks and establishing appropriate behaviors in adult life. Recent work has highlighted sex differences in the maturation processes of astrocytes during early postnatal developmental stages, showing that astrocytes in male mice reach a mature state earlier than in female mice [210]. Sex-dependent maturational trajectories are influenced by the perinatal surge in testosterone aimed at masculinizing the brain after birth and establishing sexually dimorphic brain circuitries responsible for sex-differentiated behaviors and reproductive processes [211]. Astroglia respond to circulating gonadal hormones [212], which influence their relative sex-dependent maturation rates [210]. These may in turn correlate with sex-dependent closure times for temporal windows of high plasticity necessary for astrocyte-mediated modulation of neuronal circuit development. It would be highly interesting to investigate whether in ND diseases, for example, a further earlier closure of the critical period, accompanied by a putative premature differentiation of astrocytes, may account for the observed prevalence of disorders such as ASD in male individuals. Although this hypothesis has not yet been tested, we may speculate that a shorter time window for astrocyte maturation in males might make them more vulnerable to diseases affecting the astrocyte-dependent early-onset synaptic changes. This would explain some phenotypic effects seen in ASD. In such a case, there would be a narrower time frame for male individuals carrying those affected cells to recover from (or adapt to) detrimental environmental challenges occurring during this period and to rescue impaired neuronal circuits (Fig. 4). Correspondingly, slower astrocyte maturation time frames in females might trigger the onset of female-biased disorders eventually characterized by an inappropriately longer immature brain state. Similar considerations can be true for other pathologies for which a sexual dimorphism has been already recognized, opening alternative lines for their investigations [213] (Fig. 4).

## Conclusions

In conclusion, we propose astrocytes as critical novel targets for the development of efficacious medical treatments for NP and ND disorders. These disorders could potentially result from aberrant opening/closure of critical developmental periods, accompanied by defective astrocyte proliferation/maturation processes either affecting synapse formation and gyrification and/or destabilizing neuronal circuits with E/I imbalances and/or impairing an astrocyte-dependent synapse elimination. These hypothesis point out the urge of intensifying research efforts to identify the molecular drivers of such processes and propose astrocyte-based therapeutic approaches to reverse disease phenotypes. Moreover, specific attention to early postnatal brain developmental periods, in combination with the study of sex-specific differential maturation patterns, may improve our knowledge regarding physiological and pathological processes taking place during these time spans and develop treatments personalized to the needs of female and male patients.

## References

[1] Bandeira F, Lent R, Herculano-Houzel S. Changing numbers of neuronal and non-neuronal cells underlie postnatal brain growth in the rat. Proc Natl Acad Sci. 2009;106:14108–13. 19666520 10.1073/pnas.0804650106 PMC2729028

[2] Khakh BS, Sofroniew MV. Diversity of astrocyte functions and phenotypes in neural circuits. Nat Neurosci. 2015;18:942–52. 26108722 10.1038/nn.4043 PMC5258184

[3] Semyanov A, Verkhratsky A. Astrocytic processes: from tripartite synapses to the active milieu. Trends Neurosci. 2021;44:781–92. 34479758 10.1016/j.tins.2021.07.006

[4] Magistretti PJ, Allaman I. Lactate in the brain: from metabolic end-product to signalling molecule. Nat Rev Neurosci. 2018;19:235–49. 29515192 10.1038/nrn.2018.19

[5] Allen NJ, Eroglu C. Cell Biology of Astrocyte-Synapse Interactions. Neuron. 2017;96:697–708. 29096081 10.1016/j.neuron.2017.09.056 PMC5687890

[6] Bosworth AP, Allen NJ. The diverse actions of astrocytes during synaptic development. Curr Opin Neurobiol. 2017;47:38–43. 28938161 10.1016/j.conb.2017.08.017

[7] Faulkner JR, Herrmann JE, Woo MJ, Tansey KE, Doan NB, Sofroniew MV. Reactive Astrocytes Protect Tissue and Preserve Function after Spinal Cord Injury. J Neurosci. 2004;24:2143–55. 14999065 10.1523/JNEUROSCI.3547-03.2004 PMC6730429

[8] Iram T, Frenkel D. Targeting the Role of Astrocytes in the Progression of Alzheimers Disease. Curr Signal Transduct Ther. 2012;7:20–7.

[9] Molofsky AV, Kelley KW, Tsai HH, Redmond SA, Chang SM, Madireddy L, et al. Astrocyte-encoded positional cues maintain sensorimotor circuit integrity. Nature 2014;509:189–94. 24776795 10.1038/nature13161 PMC4057936

[10] Cabezas R, Ávila M, Gonzalez J, El-Bachá RS, Báez E, García-Segura LM, et al. Astrocytic modulation of blood brain barrier: perspectives on Parkinson´s disease. Front Cell Neurosci. 2014;8. Available from: http://journal.frontiersin.org/article/10.3389/fncel.2014.00211/abstract. 10.3389/fncel.2014.00211 PMC4120694 25136294

[11] Boulay AC, Saubaméa B, Adam N, Chasseigneaux S, Mazaré N, Gilbert A, et al. Translation in astrocyte distal processes sets molecular heterogeneity at the gliovascular interface. Cell Discov. 2017;3:17005. 28377822 10.1038/celldisc.2017.5 PMC5368712

[12] Alvarez JI, Katayama T, Prat A. Glial influence on the blood brain barrier. Glia. 2013;61:1939–58. 24123158 10.1002/glia.22575 PMC4068281

[13] Eroglu C, Barres BA. Regulation of synaptic connectivity by glia. Nature. 2010;468:223–31. 21068831 10.1038/nature09612 PMC4431554

[14] Vasile F, Dossi E, Rouach N. Human astrocytes: structure and functions in the healthy brain. Brain Struct Funct. 2017;222:2017–29. 28280934 10.1007/s00429-017-1383-5 PMC5504258

[15] Oberheim NA, Takano T, Han X, He W, Lin JHC, Wang F, et al. Uniquely Hominid Features of Adult Human Astrocytes. J Neurosci. 2009;29:3276–87. 19279265 10.1523/JNEUROSCI.4707-08.2009 PMC2819812

[16] Mohn TC, Koob AO. Adult Astrogenesis and the Etiology of Cortical Neurodegeneration. J Exp Neurosci. 2015;9s2:JEN.S25520. 10.4137/JEN.S25520 PMC4634839 26568684

[17] Verkhratsky A, Nedergaard M. Physiology of Astroglia. Physiol Rev. 2018;98:239–389. 29351512 10.1152/physrev.00042.2016 PMC6050349

[18] Verkhratsky A, Bush N, Nedergaard M, Butt A. The Special Case of Human Astrocytes. Neuroglia. 2018;1:21–9.

[19] Götz M, Huttner WB. The cell biology of neurogenesis. Nat Rev Mol Cell Biol. 2005;6:777–88. 16314867 10.1038/nrm1739

[20] Takouda J, Katada S, Nakashima K. Emerging mechanisms underlying astrogenesis in the developing mammalian brain. Proc Jpn Acad Ser B 2017;93:386–98. 28603210 10.2183/pjab.93.024 PMC5709539

[21] Ever L, Gaiano N. Radial ‘glial’ progenitors: neurogenesis and signaling. Curr Opin Neurobiol. 2005;15:29–33. 15721741 10.1016/j.conb.2005.01.005

[22] Zhou CJ, Zhao C, Pleasure SJ. Wnt Signaling Mutants Have Decreased Dentate Granule Cell Production and Radial Glial Scaffolding Abnormalities. J Neurosci. 2004;24:121–6. 14715945 10.1523/JNEUROSCI.4071-03.2004 PMC6729560

[23] Rowitch DH, Kriegstein AR. Developmental genetics of vertebrate glial–cell specification. Nature 2010;468:214–22. 21068830 10.1038/nature09611

[24] Eze UC, Bhaduri A, Haeussler M, Nowakowski TJ, Kriegstein AR. Single-cell atlas of early human brain development highlights heterogeneity of human neuroepithelial cells and early radial glia. Nat Neurosci. 2021;24:584–94. 33723434 10.1038/s41593-020-00794-1 PMC8012207

[25] Pollen AA, Nowakowski TJ, Chen J, Retallack H, Sandoval-Espinosa C, Nicholas CR, et al. Molecular Identity of Human Outer Radial Glia during Cortical Development. Cell. 2015;163:55–67. 26406371 10.1016/j.cell.2015.09.004 PMC4583716

[26] Miller FD, Gauthier AS. Timing is everything: making neurons versus glia in the developing cortex. Neuron. 2007;54:357–69. 17481390 10.1016/j.neuron.2007.04.019

[27] Morrow T, Song MR, Ghosh A. Sequential specification of neurons and glia by developmentally regulated extracellular factors. Dev Camb Engl. 2001;128:3585–94. 10.1242/dev.128.18.3585 11566862

[28] Ge WP, Miyawaki A, Gage FH, Jan YN, Jan LY. Local generation of glia is a major astrocyte source in postnatal cortex. Nature. 2012;484:376–80. 22456708 10.1038/nature10959 PMC3777276

[29] De Zeeuw CI, Hoogland TM. Reappraisal of Bergmann glial cells as modulators of cerebellar circuit function. Front Cell Neurosci. 2015;9. Available from: http://journal.frontiersin.org/Article/10.3389/fncel.2015.00246/abstract. 10.3389/fncel.2015.00246 PMC4488625 26190972

[30] Güngör Kobat S. Importance of Müller Cells. Beyoglu Eye J. 2020; Available from: http://beyoglueye.com/jvi.aspx?un=BEJ-28290&volume=. 10.14744/bej.2020.28290 PMC8784480 35098065

[31] Choi BH, Lapham LW. Evolution of Bergman glia in developing human fetal cerebellum: A Golgi, electron microscopic and immunofluorescent study. Brain Res. 1980;190:369–83. 6989450 10.1016/0006-8993(80)90280-2

[32] Reichenbach A, Bringmann A. Glia of the human retina. Glia. 2020;68:768–96. 31793693 10.1002/glia.23727

[33] Martinotti F. Contributo allo studio della corteccia cerebrale, ed all ´origine centrale dei nervi. Fratelli Bocca; 1889.

[34] Andriezen WL. The Neuroglia Elements in the Human Brain. BMJ. 1893;2:227–30. 20754383 10.1136/bmj.2.1700.227 PMC2422013

[35] Retzius G. Die neuroglia des Gehirns beim Menschen und bei Saeugethieren. Jena: Chemie; 1894.

[36] Colombo JA, Yáñez A, Puissant V, Lipina S. Long, interlaminar astroglial cell processes in the cortex of adult monkeys. J Neurosci Res. 1995;40:551–6. 7616615 10.1002/jnr.490400414

[37] Colombo JA. Interlaminar Astroglial Processes in the Cerebral Cortex of Adult Monkeys But Not of Adult Rats. Cells Tissues Organs. 1996;155:57–62. 10.1159/000147790 8811116

[38] Colombo JA, Yáñez A, Lipina SJ. Interlaminar astroglial processes in the cerebral cortex of non human primates: response to injury. J Hirnforsch. 1997;38:503–12. 9476215

[39] Colombo JA, Reisin HD. Interlaminar astroglia of the cerebral cortex: a marker of the primate brain. Brain Res. 2004;1006:126–31. 15047031 10.1016/j.brainres.2004.02.003

[40] Falcone C, Penna E, Hong T, Tarantal AF, Hof PR, Hopkins WD, et al. Cortical Interlaminar Astrocytes Are Generated Prenatally, Mature Postnatally, and Express Unique Markers in Human and Nonhuman Primates. Cereb Cortex. 2021;31:379–95. 32930323 10.1093/cercor/bhaa231 PMC7947181

[41] Falcone C, Martínez-Cerdeño V. Astrocyte evolution and human specificity. Neural Regen Res. 2023;18:131. 35799529 10.4103/1673-5374.340405 PMC9241407

[42] Falcone C, McBride EL, Hopkins WD, Hof PR, Manger PR, Sherwood CC, et al. Redefining varicose projection astrocytes in primates. Glia. 2022;70:145–54. 34533866 10.1002/glia.24093

[43] Han X, Chen M, Wang F, Windrem M, Wang S, Shanz S, et al. Forebrain Engraftment by Human Glial Progenitor Cells Enhances Synaptic Plasticity and Learning in Adult Mice. Cell Stem Cell. 2013;12:342–53. 23472873 10.1016/j.stem.2012.12.015 PMC3700554

[44] Azevedo FAC, Carvalho LRB, Grinberg LT, Farfel JM, Ferretti REL, Leite REP, et al. Equal numbers of neuronal and nonneuronal cells make the human brain an isometrically scaled-up primate brain. J Comp Neurol. 2009;513:532–41. 19226510 10.1002/cne.21974

[45] Falcone C. Evolution of astrocytes: From invertebrates to vertebrates. Front Cell Dev Biol. 2022;10:931311. 36046339 10.3389/fcell.2022.931311 PMC9423676

[46] Degl’Innocenti E, Dell’Anno MT. Human and mouse cortical astrocytes: a comparative view from development to morphological and functional characterization. Front Neuroanat. 2023;17:1130729. 37139179 10.3389/fnana.2023.1130729 PMC10150887

[47] Namba T, Huttner WB. Neural progenitor cells and their role in the development and evolutionary expansion of the neocortex. WIREs Dev Biol. 2017;6:e256. 10.1002/wdev.256 27865053

[48] Shinmyo Y, Saito K, Hamabe-Horiike T, Kameya N, Ando A, Kawasaki K, et al. Localized astrogenesis regulates gyrification of the cerebral cortex. Sci Adv. 2022;8:eabi5209. 35275722 10.1126/sciadv.abi5209 PMC8916738

[49] Zilles K, Palomero-Gallagher N, Amunts K. Development of cortical folding during evolution and ontogeny. Trends Neurosci. 2013;36:275–84. 23415112 10.1016/j.tins.2013.01.006

[50] Farmer WT, Murai K. Resolving Astrocyte Heterogeneity in the CNS. Front Cell Neurosci. 2017;11:300. 29021743 10.3389/fncel.2017.00300 PMC5623685

[51] Holt MG. Astrocyte heterogeneity and interactions with local neural circuits. Essays Biochem. 2023;67:93–106. 10.1042/EBC20220136 PMC10011406 36748397

[52] Miller SJ. Astrocyte Heterogeneity in the Adult Central Nervous System. Front Cell Neurosci. 2018;12:401. 30524236 10.3389/fncel.2018.00401 PMC6262303

[53] Oliveria JP, Li ZJ. critical role of astrogenesis and neurodevelopment in Fragile X Syndrome and Rett Syndrome. McMaster Univ Med J. 2020;17. Available from: https://journals.mcmaster.ca/mumj/article/view/2338.

[54] Kanski R, Van Strien ME, Van Tijn P, Hol EM. A star is born: new insights into the mechanism of astrogenesis. Cell Mol Life Sci. 2014;71:433–47. 23907612 10.1007/s00018-013-1435-9 PMC11113452

[55] Yoon K, Nery S, Rutlin ML, Radtke F, Fishell G, Gaiano N. Fibroblast Growth Factor Receptor Signaling Promotes Radial Glial Identity and Interacts with Notch1 Signaling in Telencephalic Progenitors. J Neurosci. 2004;24:9497–506. 15509736 10.1523/JNEUROSCI.0993-04.2004 PMC6730142

[56] Fan G, Martinowich K, Chin MH, He F, Fouse SD, Hutnick L, et al. DNA methylation controls the timing of astrogliogenesis through regulation of JAK-STAT signaling. Development. 2005;132:3345–56. 16014513 10.1242/dev.01912

[57] Sun Y, Nadal-Vicens M, Misono S, Lin MZ, Zubiaga A, Hua X, et al. Neurogenin Promotes Neurogenesis and Inhibits Glial Differentiation by Independent Mechanisms. Cell. 2001;104:365–76. 11239394 10.1016/s0092-8674(01)00224-0

[58] Bonni A, Sun Y, Nadal-Vicens M, Bhatt A, Frank DA, Rozovsky I, et al. Regulation of Gliogenesis in the Central Nervous System by the JAK-STAT Signaling Pathway. Science. 1997;278:477–83. 9334309 10.1126/science.278.5337.477

[59] Nieto M, Schuurmans C, Britz O, Guillemot F. Neural bHLH Genes Control the Neuronal versus Glial Fate Decision in Cortical Progenitors. Neuron. 2001;29:401–13. 11239431 10.1016/s0896-6273(01)00214-8

[60] Zhang Y, Pak C, Han Y, Ahlenius H, Zhang Z, Chanda S, et al. Rapid Single-Step Induction of Functional Neurons from Human Pluripotent Stem Cells. Neuron. 2013;78:785–98. 23764284 10.1016/j.neuron.2013.05.029 PMC3751803

[61] Yang N, Chanda S, Marro S, Ng YH, Janas JA, Haag D, et al. Generation of pure GABAergic neurons by transcription factor programming. Nat Methods. 2017;14:621–8. 28504679 10.1038/nmeth.4291 PMC5567689

[62] Vierbuchen T, Ostermeier A, Pang ZP, Kokubu Y, Südhof TC, Wernig M. Direct conversion of fibroblasts to functional neurons by defined factors. Nature. 2010;463:1035–41. 20107439 10.1038/nature08797 PMC2829121

[63] Hatakeyama J, Bessho Y, Katoh K, Ookawara S, Fujioka M, Guillemot F, et al. *Hes* genes regulate size, shape and histogenesis of the nervous system by control of the timing of neural stem cell differentiation. Development. 2004;131:5539–50. 15496443 10.1242/dev.01436

[64] Mizutani KI, Saito T. Progenitors resume generating neurons after temporary inhibition of neurogenesis by Notch activation in the mammalian cerebral cortex. Development. 2005;132:1295–304. 15750183 10.1242/dev.01693

[65] Hoch RV, Rubenstein JLR, Pleasure S. Genes and signaling events that establish regional patterning of the mammalian forebrain. Semin Cell Dev Biol. 2009;20:378–86. 19560042 10.1016/j.semcdb.2009.02.005

[66] Leone DP, Srinivasan K, Chen B, Alcamo E, McConnell SK. The determination of projection neuron identity in the developing cerebral cortex. Curr Opin Neurobiol. 2008;18:28–35. 18508260 10.1016/j.conb.2008.05.006 PMC2483251

[67] Guillemot F. Cellular and molecular control of neurogenesis in the mammalian telencephalon. Curr Opin Cell Biol. 2005;17:639–47. 16226447 10.1016/j.ceb.2005.09.006

[68] He F, Ge W, Martinowich K, Becker-Catania S, Coskun V, Zhu W, et al. A positive autoregulatory loop of Jak-STAT signaling controls the onset of astrogliogenesis. Nat Neurosci. 2005;8:616–25. 15852015 10.1038/nn1440 PMC4222251

[69] Tomita K. Mammalian achaete-scute and atonal homologs regulate neuronal versus glial fate determination in the central nervous system. EMBO J. 2000;19:5460–72. 11032813 10.1093/emboj/19.20.5460 PMC314003

[70] Llorca A, Deogracias R. Origin, Development, and Synaptogenesis of Cortical Interneurons. Front Neurosci. 2022;16:929469. 35833090 10.3389/fnins.2022.929469 PMC9272671

[71] Styr B, Slutsky I. Imbalance between firing homeostasis and synaptic plasticity drives early-phase Alzheimer’s disease. Nat Neurosci. 2018;21:463–73. 29403035 10.1038/s41593-018-0080-x PMC6533171

[72] Rubenstein JLR, Merzenich MM. Model of autism: increased ratio of excitation/inhibition in key neural systems. Genes Brain Behav. 2003;2:255–67. 14606691 10.1034/j.1601-183x.2003.00037.x PMC6748642

[73] Foss-Feig JH, Adkinson BD, Ji JL, Yang G, Srihari VH, McPartland JC, et al. Searching for Cross-Diagnostic Convergence: Neural Mechanisms Governing Excitation and Inhibition Balance in Schizophrenia and Autism Spectrum Disorders. Biol Psychiatry. 2017;81:848–61. 28434615 10.1016/j.biopsych.2017.03.005 PMC5436134

[74] Barnabé-Heider F, Wasylnka JA, Fernandes KJL, Porsche C, Sendtner M, Kaplan DR, et al. Evidence that Embryonic Neurons Regulate the Onset of Cortical Gliogenesis via Cardiotrophin-1. Neuron. 2005;48:253–65. 16242406 10.1016/j.neuron.2005.08.037

[75] Farmer WT, Abrahamsson T, Chierzi S, Lui C, Zaelzer C, Jones EV, et al. Neurons diversify astrocytes in the adult brain through sonic hedgehog signaling. Science. 2016;351:849–54. 26912893 10.1126/science.aab3103

[76] Voss AJ, Lanjewar SN, Sampson MM, King A, Hill EJ, Sing A, et al. Identification of ligand-receptor pairs that drive human astrocyte development. Nat Neurosci. 2023;26:1339–51. 37460808 10.1038/s41593-023-01375-8 PMC11046429

[77] Bayraktar OA, Bartels T, Holmqvist S, Kleshchevnikov V, Martirosyan A, Polioudakis D, et al. Astrocyte layers in the mammalian cerebral cortex revealed by a single-cell in situ transcriptomic map. Nat Neurosci. 2020;23:500–9. 32203496 10.1038/s41593-020-0602-1 PMC7116562

[78] Berardi N, Pizzorusso T, Maffei L. Critical periods during sensory development. Curr Opin Neurobiol. 2000;10:138–45. 10679428 10.1016/s0959-4388(99)00047-1

[79] Chugani HT. A Critical Period of Brain Development: Studies of Cerebral Glucose Utilization with PET. Prev Med. 1998;27:184–8. 9578992 10.1006/pmed.1998.0274

[80] Sengpiel F. The critical period. Curr Biol. 2007;17:R742–3. 17803918 10.1016/j.cub.2007.06.017

[81] Knudsen EI. Sensitive Periods in the Development of the Brain and Behavior. J Cogn Neurosci. 2004;16:1412–25. 15509387 10.1162/0898929042304796

[82] Nelson CA, Gabard-Durnam LJ. Early Adversity and Critical Periods: Neurodevelopmental Consequences of Violating the Expectable Environment. Trends Neurosci. 2020;43:133–43. 32101708 10.1016/j.tins.2020.01.002 PMC8092448

[83] Virolainen SJ, VonHandorf A. Viel KCMF, Weirauch MT, Kottyan LC. Gene–environment interactions and their impact on human health. Genes Immun. 2022;24:1–11. 36585519 10.1038/s41435-022-00192-6 PMC9801363

[84] Milbocker KA, Campbell TS, Collins N, Kim S, Smith IF, Roth TL, et al. Glia-Driven Brain Circuit Refinement Is Altered by Early-Life Adversity: Behavioral Outcomes. Front Behav Neurosci. 2021;15:786234. 34924972 10.3389/fnbeh.2021.786234 PMC8678604

[85] Christopherson KS, Ullian EM, Stokes CCA, Mullowney CE, Hell JW, Agah A, et al. Thrombospondins Are Astrocyte-Secreted Proteins that Promote CNS Synaptogenesis. Cell. 2005;120:421–33. 15707899 10.1016/j.cell.2004.12.020

[86] Pfrieger FW, Barres BA. Synaptic Efficacy Enhanced by Glial Cells in Vitro. Science. 1997;277:1684–7. 9287225 10.1126/science.277.5332.1684

[87] Mauch DH, Nägler K, Schumacher S, Göritz C, Müller EC, Otto A, et al. CNS Synaptogenesis Promoted by Glia-Derived Cholesterol. Science. 2001;294:1354–7. 11701931 10.1126/science.294.5545.1354

[88] Eroglu Ç, Allen NJ, Susman MW, O’Rourke NA, Park CY, Özkan E, et al. Gabapentin Receptor α2δ-1 Is a Neuronal Thrombospondin Receptor Responsible for Excitatory CNS Synaptogenesis. Cell. 2009;139:380–92. 19818485 10.1016/j.cell.2009.09.025 PMC2791798

[89] Fossati G, Pozzi D, Canzi A, Mirabella F, Valentino S, Morini R, et al. Pentraxin 3 regulates synaptic function by inducing AMPA receptor clustering via ECM remodeling and β1‐integrin. EMBO J. 2019;38:e99529. 30396995 10.15252/embj.201899529 PMC6315291

[90] Allen NJ, Bennett ML, Foo LC, Wang GX, Chakraborty C, Smith SJ, et al. Astrocyte glypicans 4 and 6 promote formation of excitatory synapses via GluA1 AMPA receptors. Nature. 2012;486:410–4. 22722203 10.1038/nature11059 PMC3383085

[91] Diniz LP, Almeida JC, Tortelli V, Vargas Lopes C, Setti-Perdigão P, Stipursky J, et al. Astrocyte-induced Synaptogenesis Is Mediated by Transforming Growth Factor β Signaling through Modulation of d-Serine Levels in Cerebral Cortex Neurons. J Biol Chem. 2012;287:41432–45. 23055518 10.1074/jbc.M112.380824 PMC3510841

[92] Diniz LP, Tortelli V, Garcia MN, Araújo APB, Melo HM, Seixas Da Silva GS, et al. Astrocyte transforming growth factor beta 1 promotes inhibitory synapse formation via CaM kinase II signaling. Glia. 2014;62:1917–31. 25042347 10.1002/glia.22713

[93] Gómez-Casati ME, Murtie JC, Rio C, Stankovic K, Liberman MC, Corfas G. Nonneuronal cells regulate synapse formation in the vestibular sensory epithelium via erbB-dependent BDNF expression. Proc Natl Acad Sci. 2010;107:17005–10. 20837532 10.1073/pnas.1008938107 PMC2947909

[94] Shan L, Zhang T, Fan K, Cai W, Liu H. Astrocyte-Neuron Signaling in Synaptogenesis. Front Cell Dev Biol. 2021;9:680301. 34277621 10.3389/fcell.2021.680301 PMC8284252

[95] Tan CX, Burrus Lane CJ, Eroglu C. Role of astrocytes in synapse formation and maturation. In: Current Topics in Developmental Biology. Elsevier; 2021;371–407. Available from: https://linkinghub.elsevier.com/retrieve/pii/S0070215320301435. 10.1016/bs.ctdb.2020.12.010 33706922

[96] Konishi H, Koizumi S, Kiyama H. Phagocytic astrocytes: Emerging from the shadows of microglia. Glia 2022;70:1009–26. 35142399 10.1002/glia.24145 PMC9305589

[97] Juraska JM, Willing J. Pubertal onset as a critical transition for neural development and cognition. Brain Res. 2017;1654:87–94. 27060769 10.1016/j.brainres.2016.04.012 PMC5053848

[98] Huttenlocher PR, Dabholkar AS. Regional differences in synaptogenesis in human cerebral cortex. J Comp Neurol. 1997;387:167–78. 9336221 10.1002/(sici)1096-9861(19971020)387:2<167::aid-cne1>3.0.co;2-z

[99] Peter RH. Synaptic density in human frontal cortex — Developmental changes and effects of aging. Brain Res. 1979;163:195–205. 427544 10.1016/0006-8993(79)90349-4

[100] Dosenbach NUF, Nardos B, Cohen AL, Fair DA, Power JD, Church JA, et al. Prediction of Individual Brain Maturity Using fMRI. Science. 2010;329:1358–61. 20829489 10.1126/science.1194144 PMC3135376

[101] Petanjek Z, Judaš M, Šimić G, Rašin MR, Uylings HBM, Rakic P, et al. Extraordinary neoteny of synaptic spines in the human prefrontal cortex. Proc Natl Acad Sci. 2011;108:13281–6. 21788513 10.1073/pnas.1105108108 PMC3156171

[102] Antoine MW, Langberg T, Schnepel P, Feldman DE. Increased Excitation-Inhibition Ratio Stabilizes Synapse and Circuit Excitability in Four Autism Mouse Models. Neuron. 2019;101:648–61.e4. 30679017 10.1016/j.neuron.2018.12.026 PMC6733271

[103] Aida T, Yoshida J, Nomura M, Tanimura A, Iino Y, Soma M, et al. Astroglial Glutamate Transporter Deficiency Increases Synaptic Excitability and Leads to Pathological Repetitive Behaviors in Mice. Neuropsychopharmacology. 2015;40:1569–79. 25662838 10.1038/npp.2015.26 PMC4915262

[104] Ortinski PI, Dong J, Mungenast A, Yue C, Takano H, Watson DJ, et al. Selective induction of astrocytic gliosis generates deficits in neuronal inhibition. Nat Neurosci. 2010;13:584–91. 20418874 10.1038/nn.2535 PMC3225960

[105] Escartin C, Galea E, Lakatos A, O’Callaghan JP, Petzold GC, Serrano-Pozo A, et al. Reactive astrocyte nomenclature, definitions, and future directions. Nat Neurosci. 2021;24:312–25. 33589835 10.1038/s41593-020-00783-4 PMC8007081

[106] Eltokhi A, Janmaat IE, Genedi M, Haarman BCM, Sommer IEC. Dysregulation of synaptic pruning as a possible link between intestinal microbiota dysbiosis and neuropsychiatric disorders. J Neurosci Res. 2020;98:1335–69. 32239720 10.1002/jnr.24616

[107] Cardozo PL, De Lima IBQ, Maciel EMA, Silva NC, Dobransky T, Ribeiro FM. Synaptic Elimination in Neurological Disorders. Curr Neuropharmacol. 2019;17:1071–95. 31161981 10.2174/1570159X17666190603170511 PMC7052824

[108] Zhuang Y, Xu X, Li H, Niu F, Yang M, Ge Q, et al. Megf10‐related engulfment of excitatory postsynapses by astrocytes following severe brain injury. CNS Neurosci Ther. 2023;29:2873–83. 10.1111/cns.14223 PMC10493650 37081759

[109] Iram T, Ramirez-Ortiz Z, Byrne MH, Coleman UA, Kingery ND, Means TK, et al. Megf10 Is a Receptor for C1Q That Mediates Clearance of Apoptotic Cells by Astrocytes. J Neurosci. 2016;36:5185–92. 27170117 10.1523/JNEUROSCI.3850-15.2016 PMC4863057

[110] Pattwell SS, Liston C, Jing D, Ninan I, Yang RR, Witztum J, et al. Dynamic changes in neural circuitry during adolescence are associated with persistent attenuation of fear memories. Nat Commun. 2016;7:11475. 27215672 10.1038/ncomms11475 PMC4890178

[111] Honeycutt JA, Demaestri C, Peterzell S, Silveri MM, Cai X, Kulkarni P, et al. Altered corticolimbic connectivity reveals sex-specific adolescent outcomes in a rat model of early life adversity. eLife. 2020;9:e52651. 31958061 10.7554/eLife.52651 PMC7010412

[112] Karpova NN, Pickenhagen A, Lindholm J, Tiraboschi E, Kulesskaya N, Ágústsdóttir A, et al. Fear Erasure in Mice Requires Synergy Between Antidepressant Drugs and Extinction Training. Science. 2011;334:1731–4. 22194582 10.1126/science.1214592 PMC3929964

[113] Vetencourt JFM, Sale A, Viegi A, Baroncelli L, De Pasquale RF, et al. The Antidepressant Fluoxetine Restores Plasticity in the Adult Visual Cortex. Science. 2008;320:385–8. 18420937 10.1126/science.1150516

[114] Ribot J, Breton R, Calvo CF, Moulard J, Ezan P, Zapata J, et al. Astrocytes close the mouse critical period for visual plasticity. Science. 2021;373:77–81. 34210880 10.1126/science.abf5273

[115] Müller CM, Best J. Ocular dominance plasticity in adult cat visual cortex after transplantation of cultured astrocytes. Nature. 1989;342:427–30. 2586611 10.1038/342427a0

[116] Ghézali G, Calvo CF, Pillet LE, Llense F, Ezan P, Pannasch U, et al. Connexin 30 controls astroglial polarization during postnatal brain development. Development. 2018;145:dev155275. 29475972 10.1242/dev.155275 PMC5869003

[117] Abbink MR, Deijk AF, Heine VM, Verheijen MH, Korosi A. The involvement of astrocytes in early‐life adversity induced programming of the brain. Glia. 2019;67:1637–53. 10.1002/glia.23625 PMC6767561 31038797

[118] Codeluppi SA, Chatterjee D, Prevot TD, Bansal Y, Misquitta KA, Sibille E, et al. Chronic Stress Alters Astrocyte Morphology in Mouse Prefrontal Cortex. Int J Neuropsychopharmacol. 2021;24:842–53. 34346493 10.1093/ijnp/pyab052 PMC8538896

[119] Woodburn SC, Bollinger JL, Wohleb ES. Synaptic and behavioral effects of chronic stress are linked to dynamic and sex-specific changes in microglia function and astrocyte dystrophy. Neurobiol Stress. 2021;14:100312. 33748354 10.1016/j.ynstr.2021.100312 PMC7970222

[120] Dolotov OV, Inozemtseva LS, Myasoedov NF, Grivennikov IA. Stress-Induced Depression and Alzheimer’s Disease: Focus on Astrocytes. Int J Mol Sci. 2022;23:4999. 35563389 10.3390/ijms23094999 PMC9104432

[121] Naskar S, Chattarji S. Stress Elicits Contrasting Effects on the Structure and Number of Astrocytes in the Amygdala versus Hippocampus. eNeuro. 2019;6:ENEURO.0338-18.2019. 30783612 10.1523/ENEURO.0338-18.2019 PMC6378323

[122] Murphy‐Royal C, Gordon GR, Bains JS. Stress‐induced structural and functional modifications of astrocytes—Further implicating glia in the central response to stress. Glia. 2019;67:1806–20. 30889320 10.1002/glia.23610

[123] Yoshino K, Oda Y, Kimura M, Kimura H, Nangaku M, Shirayama Y, et al. The alterations of glutamate transporter 1 and glutamine synthetase in the rat brain of a learned helplessness model of depression. Psychopharmacology. 2020;237:2547–53. 32445055 10.1007/s00213-020-05555-3

[124] Tynan RJ, Beynon SB, Hinwood M, Johnson SJ, Nilsson M, Woods JJ, et al. Chronic stress-induced disruption of the astrocyte network is driven by structural atrophy and not loss of astrocytes. Acta Neuropathol. 2013;126:75–91. 23512378 10.1007/s00401-013-1102-0

[125] Virmani G, D’almeida P, Nandi A, Marathe S. Subfield‐specific effects of chronic mild unpredictable stress on hippocampal astrocytes. Eur J Neurosci. 2021;54:5730–46. 10.1111/ejn.15234 33866634

[126] Lu CL, Ren J, Mo JW, Fan J, Guo F, Chen LY, et al. Glucocorticoid Receptor–Dependent Astrocytes Mediate Stress Vulnerability. Biol Psychiatry. 2022;92:204–15. 35151464 10.1016/j.biopsych.2021.11.022

[127] Huang D, Li C, Zhang W, Qin J, Jiang W, Hu C. Dysfunction of astrocytic connexins 30 and 43 in the medial prefrontal cortex and hippocampus mediates depressive-like behaviours. Behav Brain Res. 2019;372:111950. 31103752 10.1016/j.bbr.2019.111950

[128] Byun YG, Kim NS, Kim G, Jeon YS, Choi JB, Park CW, et al. Stress induces behavioral abnormalities by increasing expression of phagocytic receptor MERTK in astrocytes to promote synapse phagocytosis. Immunity. 2023;56:2105–20.e13. 37527657 10.1016/j.immuni.2023.07.005

[129] Miguel-Hidalgo JJ, Moulana M, Deloach PH, Rajkowska G. Chronic Unpredictable Stress Reduces Immunostaining for Connexins 43 and 30 and Myelin Basic Protein in the Rat Prelimbic and Orbitofrontal Cortices. Chronic Stress. 2018;2:247054701881418. 10.1177/2470547018814186 PMC6375503 30775650

[130] Kang Y, Kang W, Kim A, Tae WS, Ham BJ, Han KM. Decreased cortical gyrification in major depressive disorder. Psychol Med. 2023;53:7512–24. 37154200 10.1017/S0033291723001216

[131] Ning M, Li C, Gao L, Fan J. Core-Symptom-Defined Cortical Gyrification Differences in Autism Spectrum Disorder. Front Psychiatry. 2021;12:619367. 33959045 10.3389/fpsyt.2021.619367 PMC8093770

[132] Takayanagi Y, Sasabayashi D, Takahashi T, Komori Y, Furuichi A, Kido M, et al. Altered brain gyrification in deficit and non-deficit schizophrenia. Psychol Med. 2019;49:573–80. 29739476 10.1017/S0033291718001228

[133] Cao B, Mwangi B, Passos IC, Wu MJ, Keser Z, Zunta-Soares GB, et al. Lifespan Gyrification Trajectories of Human Brain in Healthy Individuals and Patients with Major Psychiatric Disorders. Sci Rep. 2017;7:511. 28360420 10.1038/s41598-017-00582-1 PMC5428697

[134] Sasabayashi D, Takahashi T, Takayanagi Y, Suzuki M. Anomalous brain gyrification patterns in major psychiatric disorders: a systematic review and transdiagnostic integration. Transl Psychiatry. 2021;11:176. 33731700 10.1038/s41398-021-01297-8 PMC7969935

[135] Rajkowska G, Miguel-Hidalgo JJ. Glial Pathology in Major Depressive Disorder: An Approach to Investigate the Coverage of Blood Vessels by Astrocyte Endfeet in Human Postmortem Brain. In: Di Benedetto B, editor. Astrocytes. New York, NY: Springer New York; 2019. p. 247–54. (Methods in Molecular Biology; vol. 1938). Available from: http://link.springer.com/10.1007/978-1-4939-9068-9_17. 10.1007/978-1-4939-9068-9_17 30617985

[136] Di Benedetto B, Rupprecht R. Targeting Glia Cells: Novel Perspectives for the Treatment of Neuropsychiatric Diseases. Curr Neuropharmacol. 2013;11:171–85. 23997752 10.2174/1570159X11311020004 PMC3637671

[137] Roman C, Egert L, Di Benedetto B. Astrocytic‐neuronal crosstalk gets jammed: Alternative perspectives on the onset of neuropsychiatric disorders. Eur J Neurosci. 2021;54:5717–29. 32644273 10.1111/ejn.14900

[138] Nagy C, Suderman M, Yang J, Szyf M, Mechawar N, Ernst C, et al. Astrocytic abnormalities and global DNA methylation patterns in depression and suicide. Mol Psychiatry. 2015;20:320–8. 24662927 10.1038/mp.2014.21 PMC5293540

[139] Martins-Macedo J, Salgado AJ, Gomes ED, Pinto L. Adult brain cytogenesis in the context of mood disorders: From neurogenesis to the emergent role of gliogenesis. Neurosci Biobehav Rev. 2021;131:411–28. 34555383 10.1016/j.neubiorev.2021.09.030

[140] Feresten AH, Barakauskas V, Ypsilanti A, Barr AM, Beasley CL. Increased expression of glial fibrillary acidic protein in prefrontal cortex in psychotic illness. Schizophr Res. 2013;150:252–7. 23911257 10.1016/j.schres.2013.07.024

[141] Tarasov VV, Svistunov AA, Chubarev VN, Sologova SS, Mukhortova P, Levushkin D, et al. Alterations of Astrocytes in the Context of Schizophrenic Dementia. Front Pharm. 2020;10:1612. 10.3389/fphar.2019.01612 PMC7020441 32116664

[142] Notter T. Astrocytes in schizophrenia. Brain Neurosci Adv. 2021;5:239821282110091. 10.1177/23982128211009148 PMC8107940 33997293

[143] Vakilzadeh G, Martinez-Cerdeño V. Pathology and Astrocytes in Autism. Neuropsychiatr Dis Treat. 2023;19:841–50. 37077706 10.2147/NDT.S390053 PMC10106330

[144] Allen M, Huang BS, Notaras MJ, Lodhi A, Barrio-Alonso E, Lituma PJ, et al. Astrocytes derived from ASD individuals alter behavior and destabilize neuronal activity through aberrant Ca2+ signaling. Mol Psychiatry. 2022;27:2470–84. 35365802 10.1038/s41380-022-01486-x PMC9135629

[145] Rajkowska G, Miguel-Hidalgo J. Gliogenesis and Glial Pathology in Depression. CNS Neurol Disord - Drug Targets. 2007;6:219–33. 17511618 10.2174/187152707780619326 PMC2918806

[146] Belleau EL, Treadway MT, Pizzagalli DA. The Impact of Stress and Major Depressive Disorder on Hippocampal and Medial Prefrontal Cortex Morphology. Biol Psychiatry. 2019;85:443–53. 30470559 10.1016/j.biopsych.2018.09.031 PMC6380948

[147] Malykhin NV, Carter R, Seres P, Coupland NJ. Structural changes in the hippocampus in major depressive disorder: contributions of disease and treatment. J Psychiatry Neurosci. 2010;35:337–43. 20731966 10.1503/jpn.100002 PMC2928287

[148] Geng R, Huang X. Identification of major depressive disorder disease-related genes and functional pathways based on system dynamic changes of network connectivity. BMC Med Genomics. 2021;14:55. 33622334 10.1186/s12920-021-00908-z PMC7903654

[149] Czéh B, Simon M, Schmelting B, Hiemke C, Fuchs E. Astroglial Plasticity in the Hippocampus is Affected by Chronic Psychosocial Stress and Concomitant Fluoxetine Treatment. Neuropsychopharmacology. 2006;31:1616–26. 16395301 10.1038/sj.npp.1300982

[150] Czéh B, Di Benedetto B. Antidepressants act directly on astrocytes: Evidences and functional consequences. Eur Neuropsychopharmacol. 2013;23:171–85. 22609317 10.1016/j.euroneuro.2012.04.017

[151] Czéh B, Nagy SA. Clinical Findings Documenting Cellular and Molecular Abnormalities of Glia in Depressive Disorders. Front Mol Neurosci. 2018;11:56. 29535607 10.3389/fnmol.2018.00056 PMC5835102

[152] Papouin T, Ladépêche L, Ruel J, Sacchi S, Labasque M, Hanini M, et al. Synaptic and Extrasynaptic NMDA Receptors Are Gated by Different Endogenous Coagonists. Cell. 2012;150:633–46. 22863013 10.1016/j.cell.2012.06.029

[153] Henneberger C, Papouin T, Oliet SHR, Rusakov DA. Long-term potentiation depends on release of d-serine from astrocytes. Nature. 2010;463:232–6. 20075918 10.1038/nature08673 PMC2807667

[154] Blanco-Suarez E, Liu TF, Kopelevich A, Allen NJ. Astrocyte-Secreted Chordin-like 1 Drives Synapse Maturation and Limits Plasticity by Increasing Synaptic GluA2 AMPA Receptors. Neuron. 2018;100:1116–32.e13. 30344043 10.1016/j.neuron.2018.09.043 PMC6382071

[155] Caldwell ALM, Sancho L, Deng J, Bosworth A, Miglietta A, Diedrich JK, et al. Aberrant astrocyte protein secretion contributes to altered neuronal development in multiple models of neurodevelopmental disorders. Nat Neurosci. 2022;25:1163–78. 36042312 10.1038/s41593-022-01150-1 PMC10395413

[156] Heresco-Levy U, Javitt DC, Ebstein R, Vass A, Lichtenberg P, Bar G, et al. D-serine efficacy as add-on pharmacotherapy to risperidone and olanzapine for treatment-refractory schizophrenia. Biol Psychiatry. 2005;57:577–85. 15780844 10.1016/j.biopsych.2004.12.037

[157] Kantrowitz JT, Malhotra AK, Cornblatt B, Silipo G, Balla A, Suckow RF, et al. High dose D-serine in the treatment of schizophrenia. Schizophr Res. 2010;121:125–30. 20541910 10.1016/j.schres.2010.05.012 PMC3111070

[158] Ma TM, Abazyan S, Abazyan B, Nomura J, Yang C, Seshadri S, et al. Pathogenic disruption of DISC1-serine racemase binding elicits schizophrenia-like behavior via D-serine depletion. Mol Psychiatry. 2013;18:557–67. 22801410 10.1038/mp.2012.97 PMC3475769

[159] Cardno AG. Gottesman II. Twin studies of schizophrenia: From bow-and-arrow concordances to Star Wars Mx and functional genomics. Am J Med Genet. 2000;97:12–7. 10813800

[160] Walker EF, Trotman HD, Pearce BD, Addington J, Cadenhead KS, Cornblatt BA, et al. Cortisol Levels and Risk for Psychosis: Initial Findings from the North American Prodrome Longitudinal Study. Biol Psychiatry. 2013;74:410–7. 23562006 10.1016/j.biopsych.2013.02.016 PMC3707958

[161] Selemon LD, Zecevic N. Schizophrenia: a tale of two critical periods for prefrontal cortical development. Transl Psychiatry. 2015;5:e623. 26285133 10.1038/tp.2015.115 PMC4564568

[162] Sheu JR, Hsieh CY, Jayakumar T, Tseng MF, Lee HN, Huang SW, et al. A Critical Period for the Development of Schizophrenia-Like Pathology by Aberrant Postnatal Neurogenesis. Front Neurosci. 2019;13:635. 31275109 10.3389/fnins.2019.00635 PMC6591536

[163] De Oliveira Figueiredo EC, Calì C, Petrelli F, Bezzi P. Emerging evidence for astrocyte dysfunction in schizophrenia. Glia. 2022;70:1585–604. 35634946 10.1002/glia.24221 PMC9544982

[164] Russo FB, Freitas BC, Pignatari GC, Fernandes IR, Sebat J, Muotri AR, et al. Modeling the Interplay Between Neurons and Astrocytes in Autism Using Human Induced Pluripotent Stem Cells. Biol Psychiatry. 2018;83:569–78. 29129319 10.1016/j.biopsych.2017.09.021

[165] Berger JM, Rohn TT, Oxford JT. Autism as the Early Closure of a Neuroplastic Critical Period Normally Seen in Adolescence. Biol Syst Open Access. 2012;02. Available from: https://www.omicsgroup.org/journals/autism-as-the-early-closure-of-a-neuroplastic-critical-period-normally-seen-in-adolescence-2329-6577-1000118.php?aid=43859. 10.4172/2329-6577.1000118 PMC3864123 24353985

[166] Hashimoto Y, Greene C, Munnich A, Campbell M. The CLDN5 gene at the blood-brain barrier in health and disease. Fluids Barriers CNS. 2023;20:22. 36978081 10.1186/s12987-023-00424-5 PMC10044825

[167] Igarashi Y, Utsumi H, Chiba H, Yamada-Sasamori Y, Tobioka H, Kamimura Y, et al. Glial Cell Line-Derived Neurotrophic Factor Induces Barrier Function of Endothelial Cells Forming the Blood–Brain Barrier. Biochem Biophys Res Commun. 1999;261:108–12. 10405331 10.1006/bbrc.1999.0992

[168] Rajkowska G, Hughes J, Stockmeier CA, Javier Miguel-Hidalgo J, Maciag D. Coverage of Blood Vessels by Astrocytic Endfeet Is Reduced in Major Depressive Disorder. Biol Psychiatry. 2013;73:613–21. 23146357 10.1016/j.biopsych.2012.09.024 PMC3578083

[169] Lee E, Chung WS. Glial Control of Synapse Number in Healthy and Diseased Brain. Front Cell Neurosci. 2019;13:42. 30814931 10.3389/fncel.2019.00042 PMC6381066

[170] Di Benedetto B, Malik VA, Begum S, Jablonowski L, Gómez-González GB, Neumann ID, et al. Fluoxetine Requires the Endfeet Protein Aquaporin-4 to Enhance Plasticity of Astrocyte Processes. Front Cell Neurosci. 2016;10. Available from: http://journal.frontiersin.org/Article/10.3389/fncel.2016.00008/abstract. 10.3389/fncel.2016.00008 PMC4735422 26869881

[171] Malik VA, Zajicek F, Mittmann LA, Klaus J, Unterseer S, Rajkumar S, et al. GDF15 promotes simultaneous astrocyte remodeling and tight junction strengthening at the blood–brain barrier. J Neurosci Res. 2020;98:1433–56. 32170776 10.1002/jnr.24611

[172] Williams BP, Price J. Evidence for multiple precursor cell types in the embryonic rat cerebral cortex. Neuron. 1995;14:1181–8. 7605631 10.1016/0896-6273(95)90265-1

[173] Chung WS, Allen NJ, Eroglu C. Astrocytes Control Synapse Formation, Function, and Elimination. Cold Spring Harb Perspect Biol. 2015;7:a020370. 25663667 10.1101/cshperspect.a020370 PMC4527946

[174] Chung WS, Clarke LE, Wang GX, Stafford BK, Sher A, Chakraborty C, et al. Astrocytes mediate synapse elimination through MEGF10 and MERTK pathways. Nature. 2013;504:394–400. 24270812 10.1038/nature12776 PMC3969024

[175] Logan MA, Freeman MR. The scoop on the fly brain: glial engulfment functions in Drosophila. Neuron Glia Biol. 2007;3:63–74. 18172512 10.1017/S1740925X07000646 PMC2171361

[176] Freeman MR, Delrow J, Kim J, Johnson E, Doe CQ. Unwrapping Glial Biology. Neuron. 2003;38:567–80. 12765609 10.1016/s0896-6273(03)00289-7

[177] Reddien PW, Horvitz HR. The engulfment process of programmed cell death in *Caenorhabditis elegans*. Annu Rev Cell Dev Biol. 2004;20:193–221. 15473839 10.1146/annurev.cellbio.20.022003.114619

[178] Stevens B, Allen NJ, Vazquez LE, Howell GR, Christopherson KS, Nouri N, et al. The Classical Complement Cascade Mediates CNS Synapse Elimination. Cell. 2007;131:1164–78. 18083105 10.1016/j.cell.2007.10.036

[179] Schafer DP, Lehrman EK, Kautzman AG, Koyama R, Mardinly AR, Yamasaki R, et al. Microglia Sculpt Postnatal Neural Circuits in an Activity and Complement-Dependent Manner. Neuron. 2012;74:691–705. 22632727 10.1016/j.neuron.2012.03.026 PMC3528177

[180] Paolicelli RC, Bolasco G, Pagani F, Maggi L, Scianni M, Panzanelli P, et al. Synaptic Pruning by Microglia Is Necessary for Normal Brain Development. Science. 2011;333:1456–8. 21778362 10.1126/science.1202529

[181] Dejanovic B, Wu T, Tsai MC, Graykowski D, Gandham VD, Rose CM, et al. Complement C1q-dependent excitatory and inhibitory synapse elimination by astrocytes and microglia in Alzheimer’s disease mouse models. Nat Aging. 2022;2:837–50. 37118504 10.1038/s43587-022-00281-1 PMC10154216

[182] Favuzzi E, Huang S, Saldi GA, Binan L, Ibrahim LA, Fernández-Otero M, et al. GABA-receptive microglia selectively sculpt developing inhibitory circuits. Cell. 2021;184:4048–63.e32. 34233165 10.1016/j.cell.2021.06.018 PMC9122259

[183] Park J, Choi Y, Jung E, Lee S, Sohn J, Chung W. Microglial MERTK eliminates phosphatidylserine‐displaying inhibitory post‐synapses. EMBO J. 2021;40:e107121. 34013588 10.15252/embj.2020107121 PMC8327958

[184] Scott‐Hewitt N, Perrucci F, Morini R, Erreni M, Mahoney M, Witkowska A, et al. Local externalization of phosphatidylserine mediates developmental synaptic pruning by microglia. EMBO J. 2020;39:e105380. 32657463 10.15252/embj.2020105380 PMC7429741

[185] Schmidtner AK, Slattery DA, Gläsner J, Hiergeist A, Gryksa K, Malik VA, et al. Minocycline alters behavior, microglia and the gut microbiome in a trait-anxiety-dependent manner. Transl Psychiatry. 2019;9:223. 31519869 10.1038/s41398-019-0556-9 PMC6744405

[186] Cullheim S, Thams S. The microglial networks of the brain and their role in neuronal network plasticity after lesion. Brain Res Rev. 2007;55:89–96. 17509690 10.1016/j.brainresrev.2007.03.012

[187] Datta D, Leslie SN, Morozov YM, Duque A, Rakic P, Van Dyck CH, et al. Classical complement cascade initiating C1q protein within neurons in the aged rhesus macaque dorsolateral prefrontal cortex. J Neuroinflammation. 2020;17:8. 31906973 10.1186/s12974-019-1683-1 PMC6945481

[188] Geloso MC, D’Ambrosi N. Microglial Pruning: Relevance for Synaptic Dysfunction in Multiple Sclerosis and Related Experimental Models. Cells. 2021;10:686. 33804596 10.3390/cells10030686 PMC8003660

[189] Hammond TR, Robinton D, Stevens B. Microglia and the Brain: Complementary Partners in Development and Disease. Annu Rev Cell Dev Biol. 2018;34:523–44. 30089221 10.1146/annurev-cellbio-100616-060509

[190] Lee JH, Kim JY, Noh S, Lee H, Lee SY, Mun JY, et al. Astrocytes phagocytose adult hippocampal synapses for circuit homeostasis. Nature. 2021;590:612–7. 33361813 10.1038/s41586-020-03060-3

[191] Damisah EC, Hill RA, Rai A, Chen F, Rothlin CV, Ghosh S, et al. Astrocytes and microglia play orchestrated roles and respect phagocytic territories during neuronal corpse removal in vivo. Sci Adv. 2020;6:eaba3239. 32637606 10.1126/sciadv.aba3239 PMC7319765

[192] Eladl E, Tremblay-LeMay R, Rastgoo N, Musani R, Chen W, Liu A, et al. Role of CD47 in Hematological Malignancies. J Hematol OncolJ Hematol Oncol. 2020;13:96. 32677994 10.1186/s13045-020-00930-1 PMC7364564

[193] Lehrman EK, Wilton DK, Litvina EY, Welsh CA, Chang ST, Frouin A, et al. CD47 Protects Synapses from Excess Microglia-Mediated Pruning during Development. Neuron. 2018;100:120–34.e6. 30308165 10.1016/j.neuron.2018.09.017 PMC6314207

[194] Li J, Brickler T, Banuelos A, Marjon K, Shcherbina A, Banerjee S, et al. Overexpression of CD47 is associated with brain overgrowth and 16p11.2 deletion syndrome. Proc Natl Acad Sci. 2021;118:e2005483118. 33833053 10.1073/pnas.2005483118 PMC8053942

[195] Chu Y, Jin X, Parada I, Pesic A, Stevens B, Barres B, et al. Enhanced synaptic connectivity and epilepsy in C1q knockout mice. Proc Natl Acad Sci. 2010;107:7975–80. 20375278 10.1073/pnas.0913449107 PMC2867906

[196] Hong S, Beja-Glasser VF, Nfonoyim BM, Frouin A, Li S, Ramakrishnan S, et al. Complement and microglia mediate early synapse loss in Alzheimer mouse models. Science. 2016;352:712–6. 27033548 10.1126/science.aad8373 PMC5094372

[197] Dion-Albert L, Bandeira Binder L, Daigle B, Hong-Minh A, Lebel M, Menard C. Sex differences in the blood-brain barrier: implications for mental health. Front Neuroendocrinol. 2022;65:100989. 35271863 10.1016/j.yfrne.2022.100989

[198] Blokland GAM, Grove J, Chen CY, Cotsapas C, Tobet S, Handa R, et al. Sex-Dependent Shared and Nonshared Genetic Architecture Across Mood and Psychotic Disorders. Biol Psychiatry. 2022;91:102–17. 34099189 10.1016/j.biopsych.2021.02.972 PMC8458480

[199] Riecher-Rössler A. Sex and gender differences in mental disorders. Lancet Psychiatry. 2017;4:8–9. 27856397 10.1016/S2215-0366(16)30348-0

[200] Ramiro L, Faura J, Simats A, García-Rodríguez P, Ma F, Martín L, et al. Influence of sex, age and diabetes on brain transcriptome and proteome modifications following cerebral ischemia. BMC Neurosci. 2023;24:7. 36707762 10.1186/s12868-023-00775-7 PMC9881265

[201] Iturria-Medina Y, Adewale Q, Khan AF, Ducharme S, Rosa-Neto P, O’Donnell K, et al. Unified epigenomic, transcriptomic, proteomic, and metabolomic taxonomy of Alzheimer’s disease progression and heterogeneity. Sci Adv. 2022;8:eabo6764. 36399579 10.1126/sciadv.abo6764 PMC9674284

[202] López-Cerdán A, Andreu Z, Hidalgo MR, Grillo-Risco R, Català-Senent JF, Soler-Sáez I, et al. Unveiling sex-based differences in Parkinson’s disease: a comprehensive meta-analysis of transcriptomic studies. Biol Sex Differ. 2022;13:68. 36414996 10.1186/s13293-022-00477-5 PMC9682715

[203] Maitra M, Mitsuhashi H, Rahimian R, Chawla A, Yang J, Fiori LM, et al. Cell type specific transcriptomic differences in depression show similar patterns between males and females but implicate distinct cell types and genes. Nat Commun. 2023;14:2912. 37217515 10.1038/s41467-023-38530-5 PMC10203145

[204] Hyer MM, Phillips LL, Neigh GN. Sex Differences in Synaptic Plasticity: Hormones and Beyond. Front Mol Neurosci. 2018;11:266. 30108482 10.3389/fnmol.2018.00266 PMC6079238

[205] Rutter M, Caspi A, Moffitt TE. Using sex differences in psychopathology to study causal mechanisms: unifying issues and research strategies: Using sex differences in psychopathology to study causal mechanisms. J Child Psychol Psychiatry. 2003;44:1092–115. 14626453 10.1111/1469-7610.00194

[206] Ziemka-Nalecz M, Pawelec P, Ziabska K, Zalewska T. Sex Differences in Brain Disorders. Int J Mol Sci. 2023;24:14571. 37834018 10.3390/ijms241914571 PMC10572175

[207] Ruigrok ANV, Salimi-Khorshidi G, Lai MC, Baron-Cohen S, Lombardo MV, Tait RJ, et al. A meta-analysis of sex differences in human brain structure. Neurosci Biobehav Rev. 2014;39:34–50. 24374381 10.1016/j.neubiorev.2013.12.004 PMC3969295

[208] Knickmeyer RC, Styner M, Short SJ, Lubach GR, Kang C, Hamer R, et al. Maturational Trajectories of Cortical Brain Development through the Pubertal Transition: Unique Species and Sex Differences in the Monkey Revealed through Structural Magnetic Resonance Imaging. Cereb Cortex. 2010;20:1053–63. 19703936 10.1093/cercor/bhp166 PMC2852502

[209] Dehorter N, Del Pino I. Shifting Developmental Trajectories During Critical Periods of Brain Formation. Front Cell Neurosci. 2020;14:283. 33132842 10.3389/fncel.2020.00283 PMC7513795

[210] Rurak GM, Simard S, Freitas-Andrade M, Lacoste B, Charih F, Van Geel A, et al. Sex differences in developmental patterns of neocortical astroglia: A mouse translatome database. Cell Rep. 2022;38:110310. 35108542 10.1016/j.celrep.2022.110310

[211] Clarkson J, Herbison AE. Hypothalamic control of the male neonatal testosterone surge. Philos Trans R Soc Lond B Biol Sci. 2016;371:20150115. 26833836 10.1098/rstb.2015.0115 PMC4785901

[212] Acaz-Fonseca E, Avila-Rodriguez M, Garcia-Segura LM, Barreto GE. Regulation of astroglia by gonadal steroid hormones under physiological and pathological conditions. Prog Neurobiol. 2016;144:5–26. 27283249 10.1016/j.pneurobio.2016.06.002

[213] Rurak GM, Woodside B, Aguilar-Valles A, Salmaso N. Astroglial cells as neuroendocrine targets in forebrain development: Implications for sex differences in psychiatric disease. Front Neuroendocrinol. 2021;60:100897. 33359797 10.1016/j.yfrne.2020.100897

